# First molecular detection and multilocus genotyping of *Enterocytozoon bieneusi* from pigs in Guangxi Zhuang Autonomous region, Southern China

**DOI:** 10.1186/s12917-025-04836-3

**Published:** 2025-06-04

**Authors:** Si-Ang Li, Yang Liu, Hui-Hong Lu, Ya-Fei Song, Meng-Jie Chu, Fei Huang, Shu-Yan Wang, Dong-Hui Zhou

**Affiliations:** 1 Key Laboratory of Fujian-Taiwan Animal Pathogen Biology, College of Animal Sciences, Fujian Agriculture and Forestry University, Fuzhou, 350002 China; 2 Guangxi Vocational University of Agriculture, Nanning, 530007 China

**Keywords:** *Enterocytozoon bieneusi*, Pigs, Prevalence, Genotype, Multilocus sequence typing, Guangxi Zhuang autonomous region

## Abstract

**Background enterocytozoon bieneusi:**

is a cosmopolitan microsporidian that infects a wide range of vertebrate and invertebrate hosts including humans, domestic animals and wild game. In this study, we determined the prevalence of *E. bieneusi* in pigs from Guangxi Zhuang Autonomous Region, China, examined the different genotypes present, and assessed their zoonotic potential.

**Methods:**

This study investigate the prevalence and multilocus genotyping of *E. bieneusi* in pigs from Guangxi Zhuang Autonomous Region in China. We collected 721 fecal samples from pigs in four regions (Guigang, Nanning, Hezhou and Yulin). These samples were subsequently analyzed using nested PCR and multilocus sequence typing (MLST).

**Results:**

The results demonstrated that the overall prevalence of *E. bieneusi* in pigs was 24.55%, ranging from 11.48 to 43.26% among four regions. The infection rates of *E. bieneusi* in pigs of four types (breeding pigs, piglets, nursery pigs and fattening pigs) and two feeding modes (free-range farming and intensive farming) ranged from 9.71 to 42.42%, and 16.71–34.71% respectively. The results of statistical analysis revealed that there were significant differences in the prevalence of *E. bieneusi* in different regions, types and feeding modes (*P* < 0.05). Ten novel genotypes (GXP-1 to GXP-10) and 12 known genotypes of *E. bieneusi* were identified. Genotype EbpC and EbpA were the main prevalent genotypes in this study. All the identified *E. bieneusi* genotypes were clustered to zoonotic group 1 by phylogenetic analysis. Fifty-seven samples were simultaneously amplified at three microsatellite loci and one minisatellite loci, resulting in the formation of 44 distinct multilocus genotypes (MLGs).

**Conclusions:**

The present study for the first time revealed the prevalence and genotypes diversity of *E. bieneusi* in pigs from Guangxi Zhuang Autonomous Region, China, providing foundational data for the prevention and control of this parasitic disease. Moreover, the observed genotype distribution of *E. bieneusi* suggests a substantial risk of zoonotic transmission, highlighting the need for sustained surveillance and targeted intervention strategies.

**Supplementary Information:**

The online version contains supplementary material available at 10.1186/s12917-025-04836-3.

## Background

Microsporidia are unicellular eukaryotes that exclusively parasitize the intestinal cells of their hosts and can infect a diverse range of animals, including humans, domestic animals, wild animals and companion animals [1–4]. To date, microsporidia have been classified into more than 220 genera and 1,700 species, among which 17 microsporidia species have been demonstrated to be capable of infecting humans [2, 5]. Among these species, *Enterocytozoon bieneusi* is the most widely reported opportunistic pathogen, causing more than 90% of microsporidiosis in human [6–8]. *E. bieneusi* is generally infected with weakened immune system individuals, such as HIV/AIDS-infected patients, organ transplant recipients, the elderly and children [7, 9–11]. Reported studies have shown that *E. bieneusi* can widely infect with various important economic animals, including pigs, dairy cows, goats and sheep. The highest prevalence of *E. bieneusi* in animals occurs in pigs (60%), followed by goats (19.2%) and cattle (5%), whereas the prevalence of *E. bieneusi* in humans was detected in only 1% [12]. The main transmitted route of *E. bieneusi* is fecal-oral transmission. Humans or other animals become infected by ingesting food or water contaminated by spores of *E. bieneusi* [9, 13–15]. However, other routes, such as respiratory transmission via inhalation of *E. bieneusi* spores also contribute to the spread of this pathogen [16]. A previous study reported that individuals occupationally or non-occupationally exposed to urban feral pigeons for 30 min may inhale approximately 3.5 × 10³ *E. bieneusi* spores, while nearby individuals may inhale around 1.3 × 10³ spores, leading to *E. bieneusi* infection [17]. The proliferation of mature spores in the intestinal epithelial cells, leads to shortening and reduction of surface area of the intestinal villus, causing malabsorption in the host [18, 19]. In recent years, prevalence surveys and genotype identification of *E. bieneusi* in pigs have been conducted in many regions of the world for assessing the risks for its zoonotic potential [20]. Previously reported studies have shown that pigs infected with *E. bieneusi* were usually asymptomatic, but the early pigs infected with *E. bieneusi* can excrete the spores of *E. bieneusi* for life which can contaminate the environment [20–22].

The spores of *E. bieneusi*, measured only 1.1 to 1.6 by 0.7 to 1.0 μm, are exceptionally tiny, so it is a challenge to distinguish them under light microscopes during fecal detection [18]. The detection sensitivity of the electron microscopy is low and prone to false negative result [23]. In addition, Microscopy with Gram chromotrope or modified trichrome staining is the standard method for diagnosing microsporidiosis [24]. Microsporidia spores stained with these techniques appear dark purple (Gram chromotrope) or bright red (modified trichrome) [25]. However, these staining methods are not only time-consuming and the fixatives are toxic (containing mercury and chlorine), but also unable to distinguish the species of microsporidia, with the sensitivity for fecal samples limited to 64% [23, 26]. In recent years, Polymerase Chain Reaction (PCR) targeting the internal transcribed spacer (ITS) region of the small subunits of ribosomal rRNA (*SSU* rRNA) gene has been widely adopted for genotyping *E. bieneusi*. This highly polymorphic locus enables precise discrimination of morphologically indistinguishable isolates [27, 28]. The use of sensitive and discriminative molecular technique will contribute to determining the true prevalence of microsporidiosis and possibly their potential transmission source depending on species identification [28]. To date, at least 600 genotypes have been identified based on ITS gene of *E. bieneusi*, which were classified into 11 different groups (group 1–11) [29]. Currently, more than 30 studies on *E. bieneusi* in pigs have been published from 14 countries, and more than 130 ITS genotypes of *E. bieneusi* have been identified in pigs and wild boars worldwide [30]. Among the genotypes detected in pigs, 19 genotypes of them have also been detected in human samples (CHN1, Bfrmr2, CAF1, CS-1, CS-4, D, EbpA, EbpC, EbpD, H, Henan-III, Henan-IV, I, LW1, O, PigEBITS5, PigEBITS7, PigEB10, SH8) [30]. Nevertheless, the use of single marker genes alone has inherent limitations in *E. bieneusi* genotyping. In 2011, a multilocus sequence typing (MLST) tool offering a high-resolution for genotyping *E. bieneusi* was found by Feng et al. based on microsatellite (MS1, MS3 and MS7) and minisatellite (MS4) loci. The tool was used to explore genotype taxonomy and host specificity of *E. bieneusi* [31].

Since the first report of *E. bieneusi* infection in pigs in 1996 [1], similar reports have been continuously documented worldwide. So far, *E. bieneusi* has been detected from pigs in a number of countries, including China, Malaysia, Czech and Spain [20, 22, 32, 33]. While *E. bieneusi* infection has been reported in several Chinese provinces, including infection rates of 79.8% in Southwest China, 37.5% in Zhijiang, and 46.8% in Hainan [30, 34, 35], there have been few studies on the prevalence of *E. bieneusi* in pigs from Guangxi Zhuang Autonomous Region. To address this research gap, this study aims to comprehensively investigate the prevalence and genotype distribution of *E. bieneusi* from pig populations in Guangxi Zhuang Autonomous Region. Through using nested PCR technology and multilocus sequence typing (MLST) analysis to identified and characterized for *E. bieneusi*. Ultimately, we evaluate the risk of zoonotic transmission of *E. bieneusi* by phylogenetic analysis. These results help to expand the knowledge of *E. bieneusi* distribution and provide baseline data for the prevention and control of *E. bieneusi* in China.

## Materials and methods

### Study sampling

The pig’s population in the Guangxi Zhuang Autonomous Region reached 41.1 million in 2020. This study, conducted March 2021 to May 2022, a total of 721 fecal samples were collected from free-range pig farms (314) and intensive pig farms (407) in four regions of Guangxi Zhuang Autonomous Region (177 samples from Guigang, 215 samples from Nanning, 183 samples from Hezhou and 146 samples from Yulin). These samples included 175 from breeding pigs, 164 from fattening pigs, 198 from nursery pigs and 184 from piglets. All fecal samples were directly collected from each pig’s rectum or were immediately collected from the fresh feces on the ground after defecation using sterile polyethylene gloves. These samples were numbered and marked with the information about the regions, types and feeding modes of the pig, and then stored at -80 °C until DNA extraction.

### Isolation of genomic DNA

Pretreatment of samples before DNA extraction: all fecal samples were heated at 100 °C for 5 min and then frozen at -80 °C for 5 min [36]. The procedure was repeated five times. According to the instructions of the commercial E.Z.N.A^®^Stool DNA Kit (Omega Biotek Inc., Norcross, GA, USA), the genomic DNA was extracted from processed samples and then stored at 4 °C for further use.

### PCR amplification and MLST analysis

*E. bieneusi* was identified via nested PCR amplification of the ITS region of the rRNA gene with specific primers [27]. The primary PCR amplification using outer primers produced a 435-bp product, while the nested PCR with inner primers yielded a 390-bp product. Detailed PCR reaction conditions and primer sequences for both rounds of amplification are provided in Table S1. Next, ITS positive *E. bieneusi* strain samples were subjected to nested PCR for amplifying microsatellite and minisatellite targets. Nested PCR targeting the MS1, MS3, MS4, and MS7 loci generated amplicons of 675 bp (MS1), 537 bp (MS3), 885 bp (MS4), and 471 bp (MS7), respectively (Table S1) [31]. All primers were synthesized by Shangya Biotech Co., Ltd. (Fuzhou, China). A positive control (pig-derived DNA of genotype EbpC) and a negative control (reagent water without DNA) were used in all the PCR tests performed to ensure the reliability of the results. The secondary PCR products were separated using 2% agarose gel with ethidium bromide, and then visualized under UV light.

### Nucleotide sequencing and phylogenetic analysis

The secondary PCR products of *E. bieneusi*-positive samples were bidirectionally sequenced by Sangon Biotech Co., Ltd (Xiamen, China). The SeqMan in Lasergene 7.1 software package (DNASTAR Inc., USA) was used to align and assemble bidirectional sequencing results, and then used to check the accuracy of the sequencing results according to the sequence chromatogram for each strand. The processed sequences were compared online with GenBank database using Basic Local Alignment Search Tool (BLAST) (https://blast.ncbi.nlm.nih.gov) for identifying *E. bieneusi* genotypes. The phylogenetic relationship based on ITS sequences of *E. bieneusi* was established using 69 *E. bieneusi* ITS gene reference sequences with the Maximum Likelihood (ML) method under the Kimura 2 parameter model in the software MEGA 11 [37]. Further, we studied the genetic diversity of the clinical strains of *E. bieneusi* by analyzing 1 minisatellite (MS4) and 3 microsatellites (MS1, MS3, and MS7) according to previously described [31]. Then, we performed a multilocus analysis by combining the four MS sequences to define the multilocus genotypes (MLGs). Sequence alignment was performed using the MegAlign program (DNAstar 7.1, DNASTAR Inc., Madison, WI). And we obtained the tandem sequences of MS1, MS3, MS4, and MS7 through the “Concatenate Sequence” function in Phylosite version 1.2.3 software. Finally, we conducted a phylogenetic analysis of the combined nucleotide sequences by the Kimura-2 parameter model in MEGA 11 software using the Maximum likelihood method. The bootstrap value was set to 1000 for determining support for the clades of the phylogenetic tree. The representative nucleotide sequences obtained in this study were deposited in the GenBank database with accession numbers OQ943833 to OQ943842.

### Statistical analysis

SPSS 26.0 (IBM Corp., New York, NY) was used to perform statistical analysis on all data in this study. The Chi-square test was used to assess the associations between infection rates of *E. bieneusi* and individual husbandry parameters (regions, types and feeding modes). Using the binary logistic regression analysis, the odds ratio (OR) calculated with 95% confidence intervals (95% CI) was explored to measure the strength of association between *E. bieneusi* prevalence and each univariate factor (regions, types, and feeding modes) [38, 39]. Differences were considered statistically significant when the *P*-value was less than 0.05.

## Results

### Prevalence of *E. bieneusi* in pigs and risk factors

In this study, the overall prevalence of *E. bieneusi* from pigs in Guangxi Zhuang Autonomous Region was 24.55% (177/721) (Table 1). The infection rate of *E. bieneusi* in Nanning (43.26%; 93/215) was the highest, followed by Guigang (23.73%; 42/177), Hezhou (11.48%; 21/183) and Yulin (14.38%; 21/146). Infection rate was more likely to be associated with different regions, as indicated by a significant difference in prevalence (*χ*^*2*^ = 65.716, *P* < 0.05). *E. bieneusi* prevalence in different types of pigs ranged from 9.71 to 42.42%. The highest infection rates of *E. bieneusi* were found in nursery pigs (42.42%; 84/198), however, the lowest infection rates of *E. bieneusi* were observed in breeding pigs (9.71%; 17/175). The results showed that the infection rate was more likely to be associated with different growth stages of pigs, and a significant difference was observed among groups (*χ*^*2*^ = 60.131, *P* < 0.05). A statistically significant association was found between feeding mode and *E. bieneusi* prevalence (χ² = 32.000, *P* < 0.05), with pigs in free-range farming (34.71%; 109/314) showing higher infection rates than those in intensive farming (16.71%; 68/407). There were significant associations of *E. bieneusi*-positivity with regions, types and feeding modes of pigs. The highest and lowest prevalence of *E. bieneusi* were in Nanning (93/215; 43.26%) and Hezhou (21/183; 11.48%), respectively (OR = 1.919; 95% CI [1.157–3.183]). The infection rates of *E. bieneusi* in nursery pigs were significantly higher than the infection rates of *E. bieneusi* in fattening pigs (OR = 4.045; 95% CI [2.362–6.926]; *P* < 0.05). Additionally, free-range farming had 2.897 times higher risk of *E. bieneusi* infection in pigs than intensive farming (OR = 2.897; 95% CI [1.915–4.382]).

**Table 1:** Association analysis between risk factors (regions, types and feeding modes) and *Enterocytozoon bieneusi* test-positivity in this study

Risk factor	Type of sample	No. of sample	No. of the positive sample (%)	odds ratio (95% CI)	*P*-value
Regions	Hezhou	183	21 (11.48%)	0.323 (0.171–0.608)	< 0.05
	Nanning	215	93 (43.26%)	1.919 (1.157–3.183)	
	Yulin	146	21 (14.38%)	0.438 (0.227–0.847)	
	Guigang *	177	42 (23.73%)	-	
Types	Breeding pigs	175	17 (9.71%)	0.637 (0.320–1.269)	< 0.05
	Piglets	184	48 (26.09%)	1.874 (1.070–3.283)	
	Nursery pigs	198	84 (42.42%)	4.045 (2.362–6.926)	
	Fattening pigs *	164	28 (17.07%)	-	
Feeding modes	Free-range farming	314	109 (34.71%)	2.897 (1.915–4.382)	< 0.05
	Intensive farming *	407	68 (16.71%)	-	
Total		721	177 (24.55%)		

* The values were used as references when odds ratio was calculated

- Odds ratio was not available

### Genotype distribution of *E. bieneusi*

In this study, based on the ITS gene, 22 genotypes were identified among the 177 *E. bieneusi-*positive samples, including 12 known genotypes and 10 novel genotypes (GXP-1 to GXP-10) (Table 2). Of these identified 12 known genotypes, genotype EbpC (49/177, 27.68%) and EbpA (36/177, 20.34%) were the main dominant genotypes in this study, followed by CHS5 (18/177, 10.17%), PigEb4 (18/177, 10.17%), CHG19 (14/177, 7.91%), CTS3 (13/177, 7.34%), PigEBITS5 (12/177, 6.78%), CHG7 (10/177, 5.65%), KIN-1 (6/177, 3.39%), O (6/177, 3.39%), Henan-I (2/177, 1.13%) and BLC17 (1/177, 0.57%). The novel genotypes with the highest prevalence of *E. bieneusi* were GXP-2 (11/177, 6.21%), followed by GPX-1 (4/177, 2.26%), GXP-5 (4/177, 2.26%), GXP-7 (3/177, 1.70%) and GXP-6 (2/177, 1.23%). For the novel genotypes GPX-3, GPX-4, GPX-8, GPX-9 and GPX-10, the prevalence of each was 0.57% (1/177).

**Table 2:** Prevalence and genotypes of *Enterocytozoon bieneusi* in pigs from Guangxi Zhuang autonomous region, China

Risk factor	Type of sample	No. of the positive sample	Prevalence (%)	Genotype (number)
Regions	Hezhou	(21/183)	11.48	CHG19(12), EbpC(5), CHG7(2), PigEBITS5(1), EbpA(1)
	Nanning	(93/215)	43.26	EbpA(21), EbpC(25), PigEb4(13), CHS5(12), CTS3(10), PigEBITS5(4), CHG7(3), GXP-5(3), O(3), GXP-7(3), CHG19(2), Henan-I(2), GXP-1(1), GXP-2(1), GXP-4(1), GXP-6(1), GXP-8(1), GXP-9(1), GXP-10(1)
	Yulin	(21/146)	14.38	EbpC(9), CHS5(6), O(3), EbpA(2), BLC17(1), GXP-1(1)
	Guiganga	(42/177)	23.73	EbPA(11), EbpC(9), GXP-2(8), KIN-1(5), CTS3(2), PigEBITS5(2), CHG7(4), CHS5(1), GXP-1(1), GXP-3(1), GXP-5(1)
Types	Breeding pigs	(17/175)	9.71	CTS3(7), EbpC(5), PigEb4(2), BLC17(1), GXP-7(1), GXP-9(1), GXP-10(1)
	Piglets	(48/184)	26.10	EbpA(11), CHG19(7), PigEb4(5), EbpC(3), Henan-I(2), CTS3(2), GXP-7(2), CHG7(1), CHG19(1), GXP-4(1), GXP-6(1), GXP-5(1)
	Nursery pigs	(84/198)	42.42	EbpC(21), EbpA(16), CHS5(15), GXP-2(11), PigEb4(7), O(6), PigEBITS5(6), KIN-1(6), CHG7(4), CTS3(3), GXP-7(2), GXP-1(2), GXP-3(1), GXP-5(1), GXP-5(1)
	Fattening pigs	(28/164)	17.10	EbpC(16), EbpA(3), CHG7(3), PigEBITS5(2), CHG19(2), CHS5(2), PigEb4(1), GXP-1(1)
Feeding modes	Free-range farming	(109/314)	34.71	EbpC(29), EbpA(25), CHG19(14), CTS3(12), PigEb4(12), CHS5(11), PigEBITS5(5), CHG7(3), O(3), GXP-5(3), GXP-7(3) Henan-I(2), GXP-6(2), GXP-4(1), GXP-1(1), GXP-2(1),
	Intensive farming	(68/407)	16.71	EbpC(19), EbpA(11), GXP-2(10), CHG7(7), KIN-1(6), PigEBITS5(6), CHS5(7), O(3), PigEb4(5), GXP-1(2), BLC7(1), CHG19(1), CTS3(1), GXP-3(1), GXP-5(1), GXP-8(1), GXP-9(1), GXP-10(1)
Total		177	35 (19.77%)	

- There was no genotypic of mixed infections in the sample type

### Phylogenetic relationship of *E. bieneusi*

**Fig. 1:**
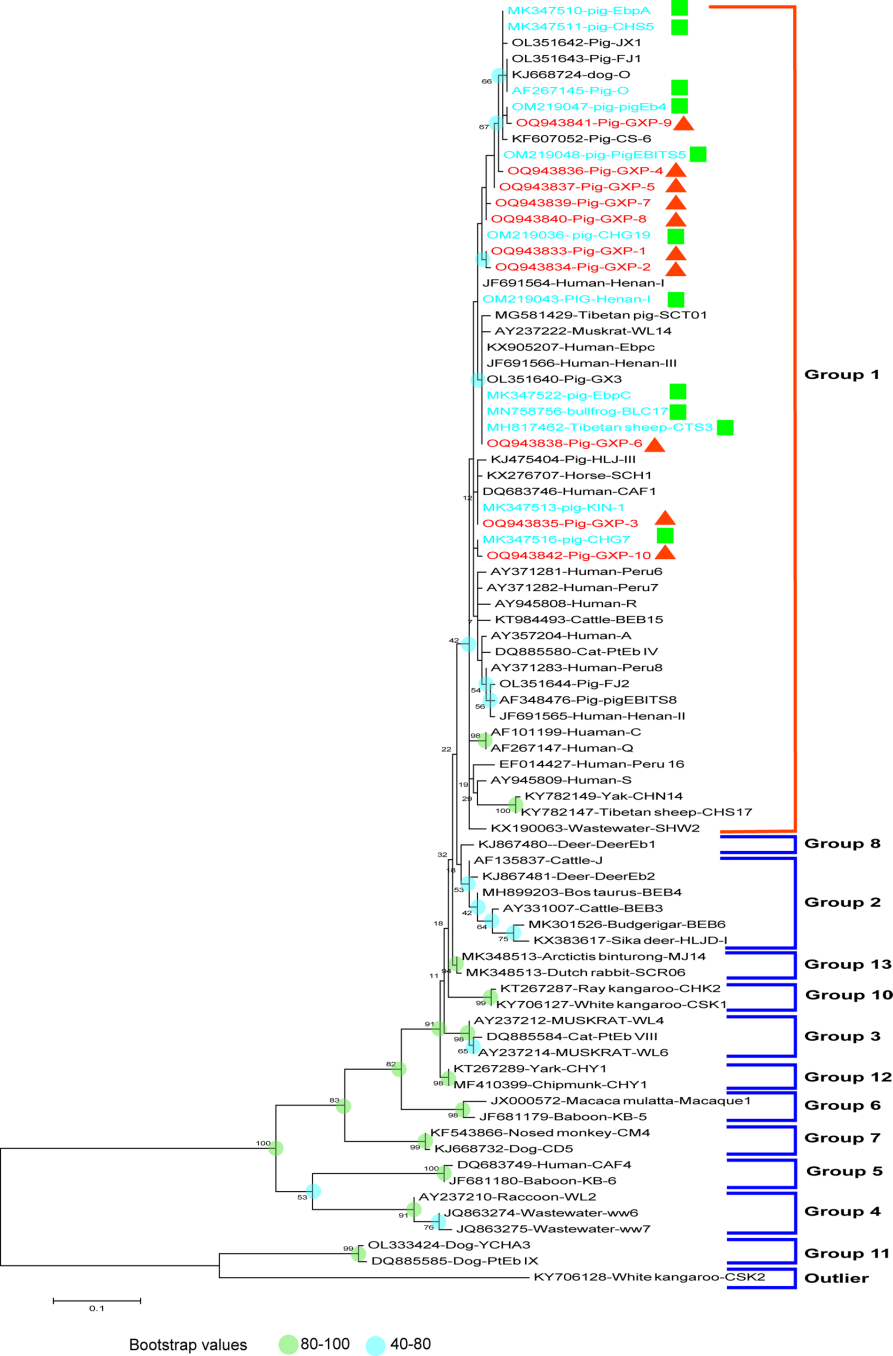
Evolutionary relationships of *E. bieneusi* groups. The relationship between the *E. bieneusi* genotypes identified in this study and other known genotypes deposited in GenBank was inferred by neighbor-joining analysis of ITS sequences based on genetic distance using the Kimura 2 parameter model. The percentage of replicate trees in which the associated taxa clustered together in the bootstrap test (1000 replicates) is shown next to the branches. The tree is drawn to scale, with branch lengths in the same units as those of the evolutionary distances used to infer the phylogenetic tree. Squares represent the known genotypes, while triangles represent the novel genotypes identified in this study

In this study, we compared the sequences of the identified novel genotypes of *E. bieneusi* with known sequences and base substitutions (G→T, G→A, A→G, C→T and T→C) at single nucleotide loci (Table 3). Single nucleotide polymorphisms (SNPs) were identified by comparing the ITS gene sequences of the 10 novel genotypes (GXP-1 to GXP-10) with GenBank database, and sequence alignment revealed similarities of was all over 99.47% for all novel genotypes. Compared with the known sequence of EbpC, GXP-1 had a G→T substitution at position 171. Similarly, GXP-2 also had a G→T substitution at position 171 and a G→A substitution at position 212. In comparison with the known sequence of KIN-1, GXP-3 had an A→G substitution at position 219. GXP-4 had a T→C substitution at position 157 compared with its homologous sequence PigEBITS5. Meanwhile, GXP-5 had an A→G substitution at position 212, and GXP-7 had a T→C substitution at position 212 and a G→A substitution at position 219. The homologous sequence of GXP-6 was CTS-3, with a homology of 99.74%, yet it had a G→T substitution at position 171. GXP-8 had the highest homology (99.74%) with the CHG19 sequence, and the substitution occurred at position 157 C→T. Compared with the known sequence of PigEB4, GXP-9 had a T→C substitution at position 251. For GXP-10, compared with its homologous sequence of CHG7, the nucleotide substitution occurred at position 164 G→A. These novel genotypes, although highly similar to the known ones in terms of sequence similarity, still exhibited some minor differences that could be crucial for understanding the genetic diversity of *E. bieneusi*. Additionally, phylogenetic analysis results indicates that these novel genotypes and 12 known genotypes (EbpC, EbpA, PigEb4, Henan-1, PigEBITS5, CHS5, CTS3, CHG7, CHG19, O, KIN-1, BLC7) identified in this study were clustered into zoonotic group 1 (Fig. 1). In the phylogenetic tree, the novel genotypes were closely clustered with some of the known genotypes, such as EbpC and PigEBITS5, suggesting a relatively close genetic relationship. The presence of novel genotypes within this group also implies that *E. bieneusi* is still evolving, and these new genotypes may represent new evolutionary directions. Epidemiologically, the fact that these genotypes belong to a zoonotic group means that there is a potential risk of cross-species transmission.

**Table 3:** Variation in nucleotide loci in the ITS sequence of *E. Bieneusi*

Genotype	Nucleotide at Position (ITS)										Homology ratio	Genbank No.
	107	157	164	171	212	217	219	251	272	344		
EbpC *	G	C	G	G	G	C	A	T	A	A		MK347522
GXP-1	-	-	-	T	-	-	-	-	-	-	99.74%	OQ943833
GXP-2	-	-	-	T	A	-	-	-	-	-	99.47%	OQ943834
KIN-1 *	G	C	G	G	G	T	A	T	A	A		MK347513
GXP-3	-	-	-	-	-	-	G	-	-	-	99.74%	OQ943835
PigEBITS5 *	A	T	G	T	A	T	G	T	A	G		OM219048
GXP-4	-	C	-	-	-	-	-	-	-	-	99.74%	OQ943836
GXP-5	-	-	-	-	G	-	-	-	-	-	99.74%	OQ943837
GXP-7	-	-	-	-	-	C	A	-	-	-	99.47%	OQ943839
CTS-3 *	G	C	G	G	G	C	A	T	A	G		MH817462
GXP-6	-	-	-	T	-	-	-	-	-	-	99.74%	OQ943838
CHG19 *	A	C	G	T	G	C	A	T	A	T		MH817463
GXP-8	-	T	-	-	-	-	-	-	-	-	99.74%	OQ943840
PigEB4 *	A	T	A	T	A	T	G	T	G	G		OM219047
GXP-9	-	-	-	-	-	-	-	C	-	-	99.74%	OQ943841
CHG7 *	A	C	G	G	G	T	G	T	A	G		MK347516
GXP-10	-	-	A	-	-	-	-	-	-	-	99.74%	OQ943842

* The genotypes were used as reference when the sequences were compared in multiple sequence comparisons

- The nucleotide sites were identical

### Multilocus sequence typing of *E. bieneusi*

**Fig. 2:**
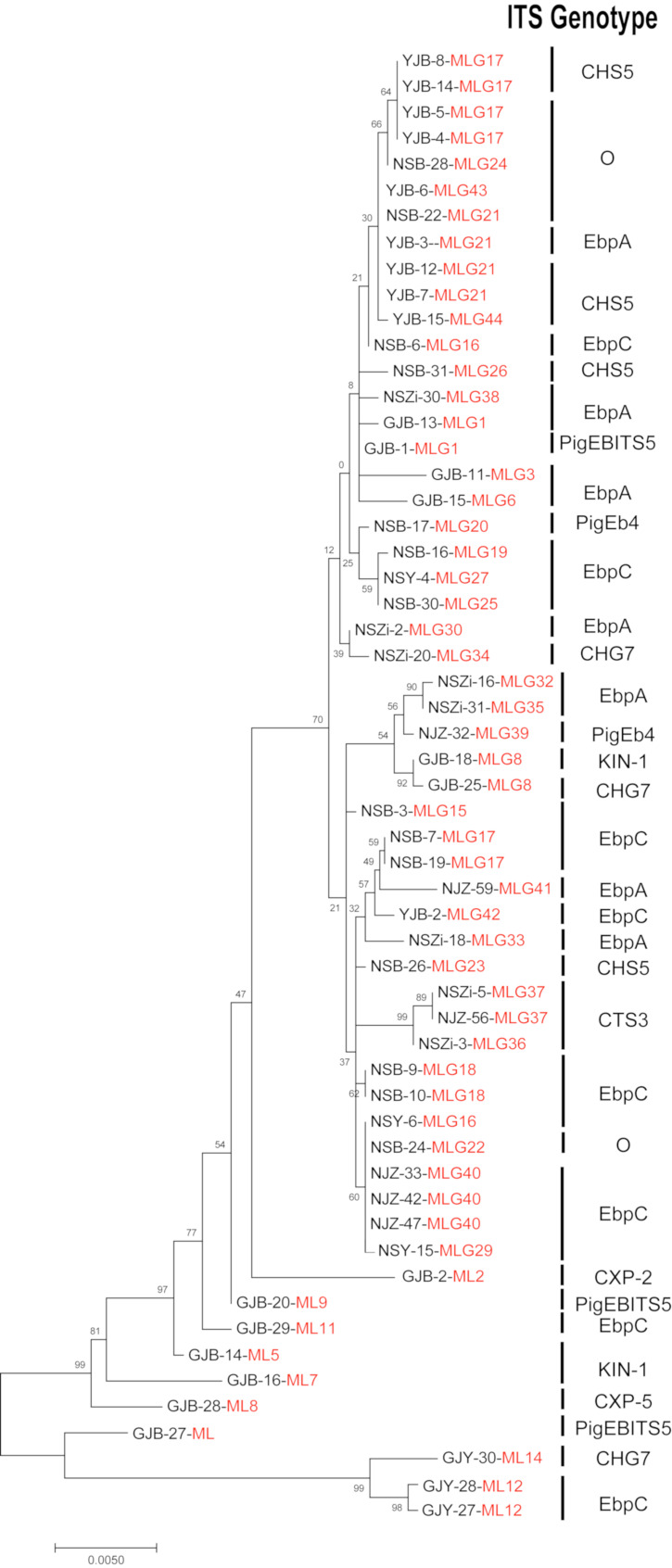
Molecular phylogenetic analysis of Enterocytozoon bieneusi strain genotypes based on concatenated sequences (MS1, MS3, MS4, and MS7) was performed using the maximum likelihood method. A maximum likelihood phylogenetic tree was constructed using MEGA 11 software (https://www.megasoftware.net) based on 57 Enterocytozoon bieneusi MLG nucleotide sequences, with the Kimura-2 parameter model and 10,000 bootstrap replications

To further explore the genetic variations of *E. bieneusi* isolates from pigs in Guangxi Zhuang Autonomous Region, a total of 177 *E. bieneusi*-positive samples were studied using the MLST technique. Out of these samples, only 57 samples successfully amplified three microsatellite loci (MS1, MS3, and MS7) and one minisatellite locus (MS4), 44 distinct MLGs (File S1 and Fig S1). The remaining samples could not amplify all four loci. There were 19, 10, 19 and 17 types of haplotypes identified at MS1, MS3, MS4 and MS7, respectively. These MLGs were distributed among 11(EbpC, EbpA, CHS5, O, KIN-1, PigEBITS5, CHG7, PigEb4, CTS3, GXP-5, GXP-2) of the 22 genotypes identified in this study. The genotype EbpC, forming 13 types of MLGs, was identified as the most diverse genotype, followed by EbpA, which included 11 types of MLGs. The analysis of evolutionary distances based on the tandem sequences of MS1, MS3, MS4, and MS7 showed that 22 genotypes were identified based on ITS. The same genotypes were also separated into different clusters (Fig. 2). These results indicated the complexity of the population structure of *E. bieneusi*, and suggested that not all individuals with the same ITS genotype belong to a closely related group. Meanwhile, it also demonstrates that these genotypes have undergone complex genetic changes during the evolutionary process.

## Discussion

The result of the present study showed an overall *E. bieneusi* prevalence of 24.55% (177/721) from pigs in Guangxi Zhuang Autonomous Region, China. Compared with the prevalence of *E. bieneusi* in pigs in other regions of China, the infection rates of this study were similar to that in Fujian Province (24.4%) [40], but lower than those reported in Shanxi Province (78.9%) [41], Xinjiang Province (48.6%) [42] and Henan Province (45.5%) [43]. When compared with the *E. bieneusi* prevalence in pigs reported from other parts of the world, the *E. bieneusi* prevalence in this study was higher than that reported from Spain (20.6%) [44], Korea (14.2%) [21], and Eastern Slovakia (5%) [45], while lower than that in Brazil (59.3%) [46], USA (31.7%) [27], and Japan (33.3%) [30, 47]. The variation in *E. bieneusi* prevalence across regions may be influenced by several factors, including sample size, sampling time, host age and feeding mode, etc. Remarkably, Vietnam is adjacent to Guangxi Zhuang Autonomous Region. In Vietnam, the zoonotic genotype E of *E. bieneusi* was detected in the feces of AIDS patients [48, 49]. The present study identified 22 *E. bieneusi* genotypes, comprising 12 known genotypes (EbpA, EbpC, CHS5, PigEb4, CHG19, CTS3, PigEBITS5, CHG7, KIN-1, O, Henan-I, BLC17) and 10 novel genotypes (GXP-1 to GXP-10). These findings underscore the necessity for both countries to adopt targeted interventions to mitigate the zoonotic transmission risk of *E. bieneusi*.

In this study, a significant difference was observed in the infection rates of *E. bieneusi* among the four regions investigated (*χ2* = 65.716, *P* < 0.05), which could be attributed to differences in feeding management techniques and breeding conditions employed in each region. In this study, the region with the highest prevalence of *E. bieneusi* was Nanning, with an infection rate of 43.26%. Nanning, the provincial capital of Guangxi Zhuang Autonomous Region, serves as a transportation hub with a high density of population and efficient circulation. However, unlike other areas in Guangxi, Nanning has a relatively small-scale swine industry. This is mainly attributed to its urbanized nature and limited farmland, which restricts large-scale agricultural expansion. In suburban districts, due to their close proximity to urban centers, pig farming predominantly relies on smallholder production systems. These small farms often lack comprehensive biosecurity measures, as many farmers have limited awareness of biosecurity protocols. As a result, the risk of exposure to pathogens increases, and these distinctive factors are likely to contribute to elevated *E. bieneusi* infection rates. The result underscores the importance of implementing effective surveillance and control measures in regions with higher infection rates, especially in areas with dense human and animal populations, as these environments present an increased risk for pathogen transmission and potential public health threats. The prevalence of *E. bieneusi* in pigs among different types exhibited significant variation (*χ2* = 60.131, *P* < 0.05). In this study, the highest infection rates of *E. bieneusi* were observed in nursery pigs among the different types of pigs, which is consistent with previous reports in pigs from southern China [50]. This result suggests a potential correlation with reduced immunity in nursery pigs. There are several factors that may lead to reduced immunity in nursery pigs. Weaning leads to a complex perceived stress in the nursery pigs, such as change in diet. The change in food from breast milk to feed may result in reduced digestibility of nursery pigs [49]. Additionally, maternal antibody levels in nursery pigs may be reduced, resulting in decreased immunity in pigs [41]. The infection rates of *E. bieneusi* also showed a statistically significant difference between different feeding modes (*χ2* = 32.000, *P* < 0.05). Free-range pigs had a 2.897 times higher risk of *E. bieneusi* infection compared to intensive pigs (OR = 2.897; 95% CI [1.915–4.382]), which may be due to the more standardized feeding management techniques and better biosafety system in intensive farms [51]. These results showed that intensive feeding mode can reduce the risk of *E. bieneusi* infection in pigs.

A total of 10 novel genotypes and 12 known genotypes were identified in this study. Single nucleotide polymorphisms (SNPs) were identified by comparing the ITS gene sequences of the 10 novel genotypes (GXP-1 to GXP-10) with GenBank database. Sequence alignment revealed similarities of these sequence were all over 99.47%. However, only 1 to 2 nucleotide site mutations occurred in these novel genotype sequences. The mutation sites were mainly concentrated at 10 different nucleotide positions. Meanwhile, the sequences comparison of the novel genotype showed a high degree of similarity to the sequences of seven genotypes reported previously (EbpC, KIN-1, PigEBITS5, CTS-3, CHG19, PigEB4, and CHG7), respectively. Nucleotide substitution, insertion, and deletion (indel) events are well - established as the primary driving forces behind genomic evolution [52]. Notably, previous studies have indicated that nucleotide substitution displays a neighboring - site preference; namely, the likelihood of a nucleotide mutation and the type of mutated nucleotide are influenced by adjacent nucleotides [52–54]. Significantly, most of the mutated sites among the sequences in this study conformed to this pattern. These results suggested that *E. bieneusi* is undergoing continuous evolution. In this study, genotypes EbpC and EbpA emerged as the most prevalent ones. Notably, our analyzed results revealed that the identified genotypes GXP-1 and GXP-2 shared a high degree of homology with EbpC. We speculate that the novel genotypes GXP-1 and GXP-2 might be new genotypes that originated from the mutation of EbpC. Moreover, genotypes EbpA and EbpC exhibit an extensive host range, encompassing non-human primates (NHPs), livestock species such as cattle, buffalo, sheep, and goats, domestic pets like dogs and horses, wild animals including deer, foxes, raccoons, bears, pandas, and otters, as well as various avian species such as pigeons, cranes, and parrots [9]. The genotype EbpC and genotype EbpA, with the prevalence of 27.68% and 20.34% respectively, were the dominant genotypes in this study, which are consistent with the results of other studies. These two genotypes have been widely reported in pigs from different regions of China [20], such as Fujian, Jilin, Henan [43], Heilongjiang [14], Zhejiang and Guangdong [55]. This indicates that genotypes EbpC and EbpA are the main prevalent genotypes in pigs in China. The high prevalence of genotypes EbpC and EbpA in China could be attributed to the following factors. The intensive pig farming practices in China create a suitable environment for the transmission and persistence of these genotypes. In such high-density farming settings, pathogens can easily spread from one animal to another [56]. The genotypes O, BLC17 and CTS3 were detected in pigs for the first time in this study, which had previously been reported in dogs [57], bullfrogs [58] and Tibetan sheep [59], respectively. This result demonstrates the potential for cross-species transmission of *E. bieneusi*. The genotypic identification of *E. bieneusi* will be further executed in these three species in Guangxi Zhuang Autonomous Region. The results of the phylogenetic analysis showed that all 22 identified genotypes were clustered into zoonotic group 1. The clustering analysis indicated that the novel genotypes identified in this study and the known ones possess zoonotic potential, which highlighting the importance of continuous surveillance and control measures to prevent possible transmission to humans.

MLST analysis has been widely used to the study of population genetics of *E. bieneusi* [60]. In this study, among 177 *E. bieneusi*-positive samples, out of 57 samples could be amplified at four loci (MS1, MS3, MS4, and MS7) through multilocus genotyping, yielding 44 different MLGs. There were 19, 10, 19 and 17 types of haplotypes identified at MS1, MS3, MS4 and MS7, respectively. These MLGs were distributed to the identified 11 genotypes (EbpC, EbpA, CHS5, O, KIN-1, PigEBITS5, CHG7, PigEb4, CTS3, GXP-5, GXP-2) in this study. In addition, Multiple MLGs were identified within the same ITS genotype of *E. bieneusi*, indicating the genetic diversity of *E. bieneusi* in pigs from Guangxi Zhuang Autonomous Region. In investigations of *E. bieneusi* in pigs from other parts of China, MLST has also been used in multilocus genotyping of *E. bieneusi*. Previously reported studies on pigs *E. bieneusi* multilocus genotyping from Fujian [40], Shaanxi [5] and Sichuan Province [61], 52, 109 and 12 positive samples amplified simultaneously at four loci formed 48, 87 and 10 MLGs, respectively. These studies clarified the high genetic diversity and the complex population structures of *E. bieneusi* in pigs. However, based on compared MLGs with ITS, a lower number of genotypes were identified by MLGs in present study. Previous study based on MLST analysis have shown that the amplification efficiencies of different loci differ substantially: MS1 has an efficiency of 50.16%, MS3 42.95%, MS4 46.61%, and MS7 41.97% [62]. Furthermore, reported research revealed that the high mutation rate in the *E. bieneusi* genome can also hinder the effective amplification of specific isolates [63]. Therefore, the development of additional dependable and efficient genetic markers is essential in the future.

## Conclusions

This study is the first to report that the infection rates of *E. bieneusi* in pigs from Guangxi Zhuang Autonomous Region were 24.55% (177/721). Among the different husbandry parameters, pigs living in Nanning, nursery pigs and free-range pigs were more likely to be infected with *E. bieneusi*. Twelve known genotypes (EbpC, EbpA, PigEb4, Henan-1, PigEBITS5, CHS5, CTS3, CHG7, CHG19, O, KIN-1 and BLC7) and ten novel genotypes (GXP-1 to GXP-10) were identified in this study, and all 22 *E. bieneusi* genotypes belonged to the zoonotic Group 1. In addition, a total of 44 distinct MLGs were identified by characterizing genetic diversity of *E. bieneusi* isolates from pigs using the MLST technique. As the first report on *E. bieneusi* prevalence and genotypes in pigs from Guangxi Zhuang Autonomous Region, this study contributes valuable knowledge to the understanding of the geographical distribution and the genetic diversity of *E. bieneusi* in pigs.

## Electronic supplementary material

Below is the link to the electronic supplementary material.

Supplementary Material 1: Fig. S1. PCR results of partial samples of ITS, MS1, MS3, MS4, and MS7 genes

Supplementary Material 2

Supplementary Material 3: File S1: Multilocus genotypes of *E. bieneusi* isolates from pigs

## Data Availability

All data generated or analyzed during this study are included in this published article. The sequence data were deposited in the GenBank database with the accession numbers: OQ943833-OQ943842.

## Abbreviations

MLST: Multilocus Sequence Typing
PCR: Polymerase Chain Reaction
ITS: Internal transcribed spacer
SSU rRNA: Small subunits of ribosomal rRNA
SSU: Small subunit
LSU: Large subunit
BLAST: Basic local alignment search tool
OR: Odds ratio
95% CI: 95% confidence intervals

## References

[1] Deplazes P, Mathis A, Muller C, Weber R. Molecular epidemiology of encephalitozoon cuniculi and first detection of *Enterocytozoon bieneusi* in faecal samples of pigs. J Eukaryot Microbiol. 1996;43(5):93S. 8822883 10.1111/j.1550-7408.1996.tb05018.x

[2] Han B, Pan G, Weiss LM. Microsporidiosis in humans. Clin Microbiol Rev. 2021;34(4):e0001020. 34190570 10.1128/CMR.00010-20 PMC8404701

[3] Kasprzak S, Topolska G. [Nosema Ceranae (Eukaryota: fungi: Microsporea)--a new parasite of Western honey bee Apis mellifera L]. Wiad Parazytol. 2007;53(4):281–4. 18441873

[4] Lom J. A catalogue of described genera and species of microsporidians parasitic in fish. Syst Parasitol. 2002;53(2):81–99. 12386417 10.1023/a:1020422209539

[5] Wang SS, Li JQ, Li YH, Wang XW, Fan XC, Liu X, Li ZJ, Song JK, Zhang LX, Zhao GH. Novel genotypes and multilocus genotypes of *Enterocytozoon bieneusi* in pigs in Northwestern China: A public health concern. Infect Genet Evol. 2018;63:89–94. 29792989 10.1016/j.meegid.2018.05.015

[6] Didier ES, Weiss LM. Microsporidiosis: current status. Curr Opin Infect Dis. 2006;19(5):485–92. 16940873 10.1097/01.qco.0000244055.46382.23 PMC3109650

[7] Mathis A, Weber R, Deplazes P. Zoonotic potential of the microsporidia. Clin Microbiol Rev. 2005;18(3):423–45. 16020683 10.1128/CMR.18.3.423-445.2005 PMC1195965

[8] Robertson LJ, Clark CG, Debenham JJ, Dubey JP, Kvac M, Li J, Ponce-Gordo F, Ryan U, Schares G, Su C, Tsaousis AD. Are molecular tools clarifying or confusing our Understanding of the public health threat from zoonotic enteric protozoa in wildlife? Int J Parasitol Parasites Wildl. 2019;9:323–41. 31338293 10.1016/j.ijppaw.2019.01.010 PMC6626983

[9] Li W, Feng Y, Santin M. Host specificity of *Enterocytozoon bieneusi* and public health implications. Trends Parasitol. 2019;35(6):436–51. 31076351 10.1016/j.pt.2019.04.004

[10] Qiu L, Xia W, Li W, Ping J, Ding S, Liu H. Author correction: the prevalence of microsporidia in China: A systematic review and meta-analysis. Sci Rep. 2019;9(1):18482. 31811164 10.1038/s41598-019-54376-8 PMC6898420

[11] Wumba R, Longo-Mbenza B, Menotti J, Mandina M, Kintoki F, Situakibanza NH, Kakicha MK, Zanga J, Mbanzulu-Makola K, Nseka T, Mukendi JP, Kendjo E, Sala J, Thellier M. Epidemiology, clinical, immune, and molecular profiles of microsporidiosis and cryptosporidiosis among HIV/AIDS patients. Int J Gen Med. 2012;5:603–11. 22924007 10.2147/IJGM.S32344 PMC3422901

[12] Udonsom R, Prasertbun R, Mahittikorn A, Chiabchalard R, Sutthikornchai C, Palasuwan A, Popruk S. Identification of *Enterocytozoon bieneusi* in goats and cattle in Thailand. BMC Vet Res. 2019;15(1):308. 31462318 10.1186/s12917-019-2054-y PMC6714406

[13] Hu Y, Feng Y, Huang C, Xiao L. Occurrence, source, and human infection potential of Cryptosporidium and *Enterocytozoon bieneusi* in drinking source water in Shanghai, China, during a pig carcass disposal incident. Environ Sci Technol. 2014;48(24):14219–27. 25383482 10.1021/es504464t PMC5788171

[14] Li W, Li Y, Li W, Yang J, Song M, Diao R, Jia H, Lu Y, Zheng J, Zhang X, Xiao L. Genotypes of *Enterocytozoon bieneusi* in livestock in China: high prevalence and zoonotic potential. PLoS ONE. 2014;9(5):e97623. 24845247 10.1371/journal.pone.0097623 PMC4028308

[15] Stentiford GD, Becnel JJ, Weiss LM, Keeling PJ, Didier ES, Williams BAP, Bjornson S, Kent ML, Freeman MA, Brown MJF, Troemel ER, Roesel K, Sokolova Y, Snowden KF, Solter LF. Microsporidia-Emergent pathogens in the global food chain (Trends in parasitology 32, 336–348; April 2, 2016). Trends Parasitol. 2016;32(8):657. 10.1016/j.pt.2016.06.002 27365191

[16] Deng L, Yue CJ, Chai YJ, Wang WY, Su XY, Zhou ZY, Wang LQ, Li LY, Liu HF, Zhong ZJ, Cao SZ, Hu YC, Fu HL, Peng GN. New genotypes and molecular characterization of *Enterocytozoon bieneusi* in pet birds in Southwestern China. Int J Parasitol Parasites Wildl. 2019;10:164–9. 31667078 10.1016/j.ijppaw.2019.08.001 PMC6811997

[17] Graczyk TK, Sunderland D, Rule AM, da Silva AJ, Moura IN, Tamang L, Girouard AS, Schwab KJ, Breysse PN. Urban feral pigeons (Columba livia) as a source for air- and waterborne contamination with *Enterocytozoon bieneusi* spores. Appl Environ Microbiol. 2007;73(13):4357–8. 17483269 10.1128/AEM.00202-07 PMC1932790

[18] Franzen C, Muller A. Molecular techniques for detection, species differentiation, and phylogenetic analysis of microsporidia. Clin Microbiol Rev. 1999;12(2):243–85. 10194459 10.1128/cmr.12.2.243 PMC88917

[19] Weber R, Sauer B, Luthy R, Nadal D. Intestinal coinfection with *Enterocytozoon bieneusi* and Cryptosporidium in a human immunodeficiency virus-infected child with chronic diarrhea. Clin Infect Dis. 1993;17(3):480–3. 8218693 10.1093/clinids/17.3.480

[20] Taghipour A, Bahadory S, Khazaei S, Zaki L, Ghaderinezhad S, Sherafati J, Abdoli A. Global molecular epidemiology of microsporidia in pigs and wild boars with emphasis on *Enterocytozoon bieneusi*: A systematic review and meta-analysis. Vet Med Sci. 2022;8(3):1126–36. 35113502 10.1002/vms3.751 PMC9122395

[21] Jeong DK, Won GY, Park BK, Hur J, You JY, Kang SJ, Oh IG, Lee YS, Stein BD, Lee JH. Occurrence and genotypic characteristics of *Enterocytozoon bieneusi* in pigs with diarrhea. Parasitol Res. 2007;102(1):123–8. 17874327 10.1007/s00436-007-0740-3

[22] Sak B, Kvac M, Hanzlikova D, Cama V. First report of *Enterocytozoon bieneusi* infection on a pig farm in the Czech Republic. Vet Parasitol. 2008;153(3–4):220–4. 18342450 10.1016/j.vetpar.2008.01.043

[23] Ahmad Ghazali WAA, Al-Talib H, Zaini AB, Microsporidiosis. Identification by a simple modified trichrome stain. Int J Infect Dis. 2020;101:430.

[24] Singh I, Sheoran AS, Zhang Q, Carville A, Tzipori S. Sensitivity and specificity of a monoclonal antibody-based fluorescence assay for detecting *Enterocytozoon bieneusi* spores in feces of Simian immunodeficiency virus-infected macaques. Clin Diagn Lab Immunol. 2005;12(10):1141–4. 16210474 10.1128/CDLI.12.10.1141-1144.2005 PMC1247839

[25] Tavalla M, Kazemi F, Kateki MM, Abdizadeh RJJJM. Molecular diagnosis of *Enterocytozoon bieneusi* and encephalitozoon spp. Wild Rats Southwest Iran. 2018;11.

[26] Datta P, Garg P, Lal Bhasin S, Malhotra P, Rana SS, Khurana S. Modified trichrome stain for faster and improved detection of intestinal protozoan parasites. Trop Doct. 2024;54(2):139–46. 38311979 10.1177/00494755241227466

[27] Buckholt MA, Lee JH, Tzipori S. Prevalence of *Enterocytozoon bieneusi* in swine: an 18-month survey at a slaughterhouse in Massachusetts. Appl Environ Microbiol. 2002;68(5):2595–9. 11976142 10.1128/AEM.68.5.2595-2599.2002 PMC127518

[28] Shehab AY, Moneer EA, Allam AF, Khalil SS, Tolba MM. Intestinal microsporidia infection in leukemic children: microscopic and molecular detection. Acta Parasitol. 2021;66(2):346–53. 32996015 10.1007/s11686-020-00283-2

[29] Zhang Y, Koehler AV, Wang T, Gasser RB. *Enterocytozoon bieneusi* of animals-With an ‘Australian twist’. Adv Parasitol. 2021;111:1–73. 33482973 10.1016/bs.apar.2020.10.001

[30] Zhou HH, Zheng XL, Ma TM, Qi M, Zhou JG, Liu HJ, Lu G, Zhao W. Molecular detection of *Enterocytozoon bieneusi* in farm-raised pigs in Hainan Province, China: infection rates, genotype distributions, and zoonotic potential. Parasite. 2020;27:12. 32129760 10.1051/parasite/2020009 PMC7055476

[31] Feng Y, Li N, Dearen T, Lobo ML, Matos O, Cama V, Xiao L. Development of a multilocus sequence typing tool for high-resolution genotyping of *Enterocytozoon bieneusi*. Appl Environ Microbiol. 2011;77(14):4822–8. 21622791 10.1128/AEM.02803-10 PMC3147401

[32] Rivero-Juarez A, Dashti A, Santin M, Koster PC, Lopez-Lopez P, Risalde MA, Garcia-Bocanegra I, Gomez-Villamandos JC, Caballero-Gomez J, Frias M, Bailo B, Ortega S, Muadica AS, Calero-Bernal R, Gonzalez-Barrio D, Rivero A, Briz V, Carmena D. Diarrhoea-causing enteric protist species in intensively and extensively Raised pigs (Sus scrofa domesticus) in Southern Spain. Part II: association with hepatitis E virus susceptibility. Transbound Emerg Dis. 2022;69(4):e1172–8. 34850588 10.1111/tbed.14408

[33] Ruviniyia K, Abdullah DA, Sumita S, Lim YAL, Ooi PT, Sharma RSK. Molecular detection of Porcine *Enterocytozoon bieneusi* infection in Peninsular Malaysia and epidemiological risk factors associated with potentially zoonotic genotypes. Parasitol Res. 2020;119(5):1663–74. 32219552 10.1007/s00436-020-06648-w

[34] Ghebremichael ST, Meng X, Yang Y, Andegiorgish AK, Wu Z, Chen J, Wei J, Li T, Bao J, Zhou Z, Pan G. First identification And coinfection detection of *Enterocytozoon bieneusi*, encephalitozoon spp., Cryptosporidium spp. And Giardia duodenalis in diarrheic pigs in Southwest China. BMC Microbiol. 2023;23(1):334. 37951859 10.1186/s12866-023-03070-x PMC10640745

[35] Zhao W, Wang Y, Xin X, Liu J, Zhang X, Yan B, Liang S. Investigating *Enterocytozoon bieneusi* in pigs farmed in Zhejiang Province, China: occurrence, genotype identification, evolutionary analysis, and zoonotic risk assessment. Vet J. 2024;306:106191. 38944378 10.1016/j.tvjl.2024.106191

[36] Hidalgo A, Melo A, Romero F, Hidalgo V, Villanueva J, Fonseca-Salamanca F. DNA extraction in Echinococcus granulosus and Taenia spp. Eggs in dogs stool samples applying thermal shock. Exp Parasitol. 2018;186:10–6. 29407715 10.1016/j.exppara.2018.01.016

[37] Tamura K, Stecher G, Kumar S. MEGA11: molecular evolutionary genetics analysis version 11. Mol Biol Evol. 2021;38(7):3022–7. 33892491 10.1093/molbev/msab120 PMC8233496

[38] Zhang Y, Koehler AV, Wang T, Robertson GJ, Bradbury RS, Gasser RB. *Enterocytozoon bieneusi* genotypes in people with Gastrointestinal disorders in Queensland and Western Australia. Infect Genet Evol. 2018;65:293–9. 30125732 10.1016/j.meegid.2018.08.006

[39] Zhang Y, Mi R, Yang J, Wang J, Gong H, Huang Y, Wang X, Han X, Zhou H, Chen Z. *Enterocytozoon bieneusi* genotypes in farmed goats and sheep in Ningxia, China. Infect Genet Evol. 2020;85:104559. 32961363 10.1016/j.meegid.2020.104559

[40] Zhang N, Wu R, Ji T, Cui LL, Cao HX, Li D, Li J, Zhang L, Huang C, Zhou DH. Molecular detection, multilocus genotyping, and population genetics of *Enterocytozoon bieneusi* in pigs in southeastern China. J Eukaryot Microbiol. 2020;67(1):107–14. 31486160 10.1111/jeu.12759

[41] Wang SS, Wang RJ, Fan XC, Liu TL, Zhang LX, Zhao GH. Prevalence and genotypes of *Enterocytozoon bieneusi* in China. Acta Trop. 2018;183:142–52. 29660311 10.1016/j.actatropica.2018.04.017

[42] Li DF, Zhang Y, Jiang YX, Xing JM, Tao DY, Zhao AY, Cui ZH, Jing B, Qi M, Zhang LX. Genotyping and zoonotic potential of *Enterocytozoon bieneusi* in pigs in Xinjiang, China. Front Microbiol. 2019;10:2401. 31695688 10.3389/fmicb.2019.02401 PMC6817468

[43] Wang H, Zhang Y, Wu Y, Li J, Qi M, Li T, Wang J, Wang R, Zhang S, Jian F, Ning C, Zhang L, Occurrence. Molecular characterization, and assessment of zoonotic risk of Cryptosporidium spp., Giardia duodenalis, and *Enterocytozoon bieneusi* in pigs in Henan, central China. J Eukaryot Microbiol. 2018;65(6):893–901. 29752883 10.1111/jeu.12634

[44] Galvan-Diaz AL, Magnet A, Fenoy S, Henriques-Gil N, Haro M, Gordo FP, Millan J, Miro G, del Aguila C, Izquierdo F. Microsporidia detection and genotyping study of human pathogenic *E. bieneusi* in animals from Spain. PLoS ONE. 2014;9(3):e92289. 24651457 10.1371/journal.pone.0092289 PMC3961313

[45] Valencakova A, Danisova O. Molecular characterization of new genotypes *Enterocytozoon bieneusi* in Slovakia. Acta Trop. 2019;191:217–20. 30586572 10.1016/j.actatropica.2018.12.031

[46] Fiuza VR, Oliveira FC, Fayer R, Santin M. First report of *Enterocytozoon bieneusi* in pigs in Brazil. Parasitol Int. 2015;64(4):18–23. 25582928 10.1016/j.parint.2015.01.002

[47] Abe N, Kimata I. Molecular survey of *Enterocytozoon bieneusi* in a Japanese Porcine population. Vector Borne Zoonotic Dis. 2010;10(4):425–7. 19725762 10.1089/vbz.2009.0039

[48] Espern A, Morio F, Miegeville M, Illa H, Abdoulaye M, Meyssonnier V, Adehossi E, Lejeune A, Cam PD, Besse B, Gay-Andrieu F. Molecular study of microsporidiosis due to *Enterocytozoon bieneusi* and encephalitozoon intestinalis among human immunodeficiency virus-infected patients from two geographical areas: Niamey, Niger, and Hanoi, Vietnam. J Clin Microbiol. 2007;45(9):2999–3002. 17634305 10.1128/JCM.00684-07 PMC2045311

[49] Lejeune A, Espern A, Phung DC, Nguyen TC, Miegeville M. [Presentation of the first *Enterocytozoon bieneusi* intestinal microsporidia case in an HIV patient, Hanoi, Vietnam]. Med Mal Infect. 2005;35(7–8):425–6. 16139460 10.1016/j.medmal.2005.06.003

[50] Li S, Zou Y, Wang P, Han RY, Wang CB, Song DP, Chen XQ. A high genetic diversity of *Enterocytozoon bieneusi* in diarrheic pigs in Southern China. Transbound Emerg Dis. 2022;69(6):3562–70. 36193011 10.1111/tbed.14719

[51] Zheng L, Duarte ME, Sevarolli Loftus A, Kim SW. Intestinal health of pigs upon weaning: challenges and nutritional intervention. Front Vet Sci. 2021;8:628258. 33644153 10.3389/fvets.2021.628258 PMC7906973

[52] Zhang Z, Gerstein M. Patterns of nucleotide substitution, insertion and deletion in the human genome inferred from pseudogenes. Nucleic Acids Res. 2003;31(18):5338–48. 12954770 10.1093/nar/gkg745 PMC203328

[53] Hess ST, Blake JD, Blake RD. Wide variations in neighbor-dependent substitution rates. J Mol Biol. 1994;236(4):1022–33. 8120884 10.1016/0022-2836(94)90009-4

[54] Bulmer M. Neighboring base effects on substitution rates in pseudogenes. Mol Biol Evol. 1986;3(4):322–9. 3444408 10.1093/oxfordjournals.molbev.a040401

[55] Zou Y, Hou JL, Li FC, Zou FC, Lin RQ, Ma JG, Zhang XX, Zhu XQ. Prevalence and genotypes of *Enterocytozoon bieneusi* in pigs in Southern China. Infect Genet Evol. 2018;66:52–6. 30218706 10.1016/j.meegid.2018.09.006

[56] WHO. The Food and Agricultural Trade Dataset. [(accessed on 22 April 2025)] Available online. https://www.fao.org/faostat/zh/#country/41

[57] Karim MR, Dong H, Yu F, Jian F, Zhang L, Wang R, Zhang S, Rume FI, Ning C, Xiao L. Genetic diversity in *Enterocytozoon bieneusi* isolates from dogs and cats in China: host specificity and public health implications. J Clin Microbiol. 2014;52(9):3297–302. 24989604 10.1128/JCM.01352-14 PMC4313153

[58] Ding H, Zhao A, Wang L, Gao N, Sun Y, Li J, Qi M. Genotypes and zoonotic potential of *Enterocytozoon bieneusi* in edible bullfrogs (Lithobates catesbeiana) in China. Int J Parasitol Parasites Wildl. 2020;11:103–7. 32051812 10.1016/j.ijppaw.2020.01.004 PMC7005328

[59] Wu Y, Chang Y, Chen Y, Zhang X, Li D, Zheng S, Wang L, Li J, Ning C, Zhang L. Occurrence and molecular characterization of Cryptosporidium spp., Giardia duodenalis, and *Enterocytozoon bieneusi* from Tibetan sheep in Gansu, China. Infect Genet Evol. 2018;64:46–51. 29894792 10.1016/j.meegid.2018.06.012

[60] Li W, Xiao L. Multilocus sequence typing and population genetic analysis of *Enterocytozoon bieneusi*: host specificity and its impacts on public health. Front Genet. 2019;10:307. 31001333 10.3389/fgene.2019.00307 PMC6454070

[61] Luo R, Xiang L, Liu H, Zhong Z, Liu L, Deng L, Liu L, Huang X, Zhou Z, Fu H, Luo Y, Peng G. First report and multilocus genotyping of *Enterocytozoon bieneusi* from Tibetan pigs in Southwestern China. Parasite. 2019;26:24. 31041895 10.1051/parasite/2019021 PMC6492536

[62] Liu X, Wu Y, Yang F, Gong B, Jiang Y, Zhou K, Cao J, Zhang W, Liu A, Shen Y. Multilocus sequence typing of *Enterocytozoon bieneusi* isolates from various mammal and bird species and assessment of population structure and substructure. Front Microbiol. 2020;11:1406. 32676063 10.3389/fmicb.2020.01406 PMC7333453

[63] Li W, Feng Y, Zhang L, Xiao L. Potential impacts of host specificity on zoonotic or interspecies transmission of *Enterocytozoon bieneusi*. Infect Genet Evol. 2019;75:104033. 31494271 10.1016/j.meegid.2019.104033

