# Supplementary material for: First molecular detection and multilocus genotyping of Enterocytozoon bieneusi from pigs in Guangxi Zhuang Autonomous region, Southern China
Source: BMC Vet Res. 2025 Jun 4;21:401. doi: 10.1186/s12917-025-04836-3 (PMC12135585; doi:10.1186/s12917-025-04836-3)
Supplement: Supplementary file 1 — Supplementary Material 1: Fig. S1. PCR results of partial samples of ITS, MS1, MS3, MS4, and MS7 genes [file 12917_2025_4836_MOESM1_ESM.pdf]

Supplementary material 1.

PCR results of partial samples of ITS, MS1, MS3, MS4, and MS7 genes. M: Marker; P: Positive control; N: Negative control. (a) 1-7: Partial samples at ITS gene; (b) MS1 gene samples; (c) MS gene samples; (d) MS4 gene samples; (e) MS7 gene samples. sample information: Hezhou (HZ), Nanning (NN), Yulin (YL)， Guigang (GG).

(a) ITS

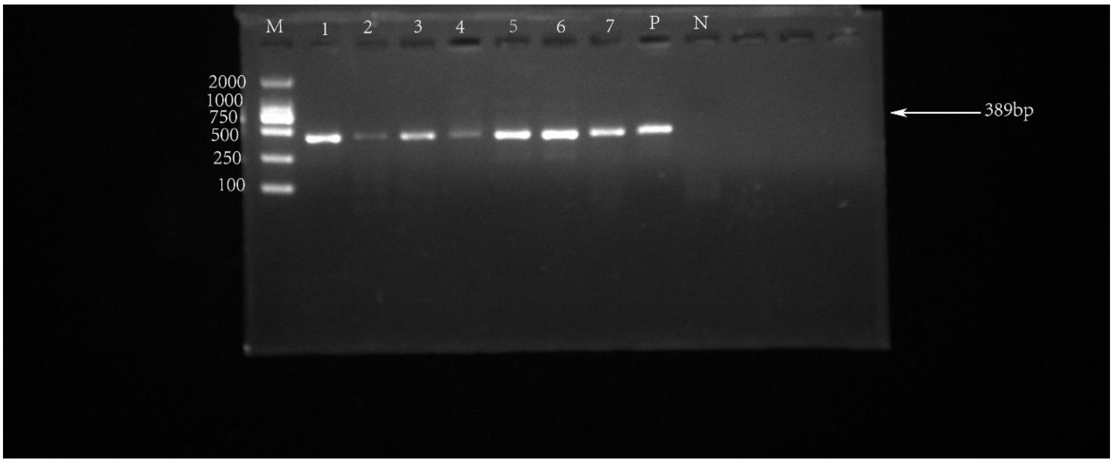

(b) MS1

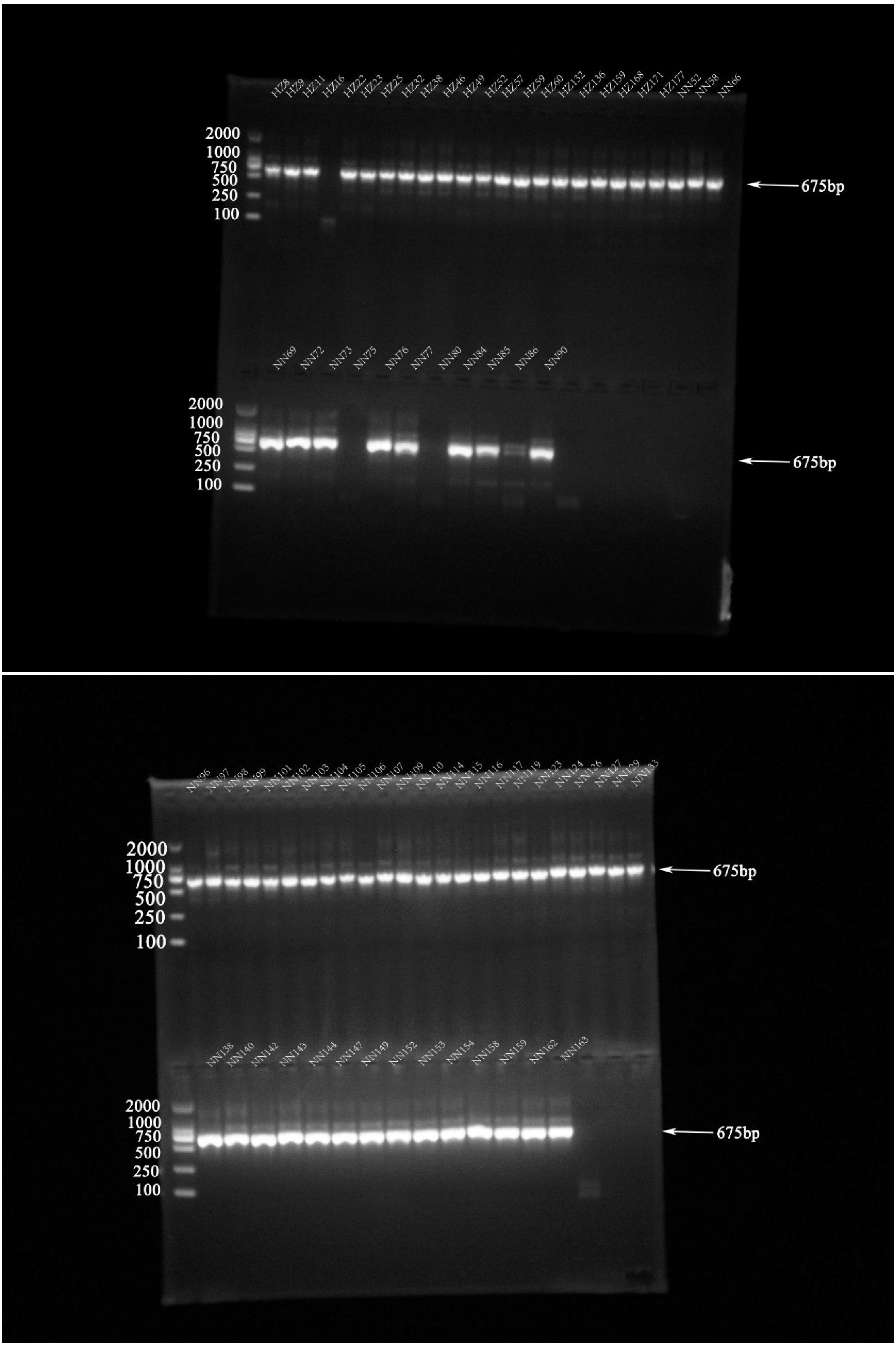
HZ8 HZ9 HZ11 HZ16 HZ22 HZ23 HZ25 HZ32 HZ38 HZ46 HZ49 HZ52 HZ57 HZ59 HZ60 HZ132 HZ136 HZ159 HZ168 HZ171 HZ177 NN52 NN58 NN66
2000
1000
750
500
250
100
675bp
NN69 NN72 NN73 NN75 NN76 NN77 NN80 NN84 NN85 NN86 NN90
2000
1000
750
500
250
100
675bp
2000
1000
750
500
250
100
675bp
NN138 NN140 NN142 NN143 NN144 NN147 NN149 NN152 NN153 NN154 NN158 NN159 NN162 NN163
2000
1000
750
500
250
100
675bp

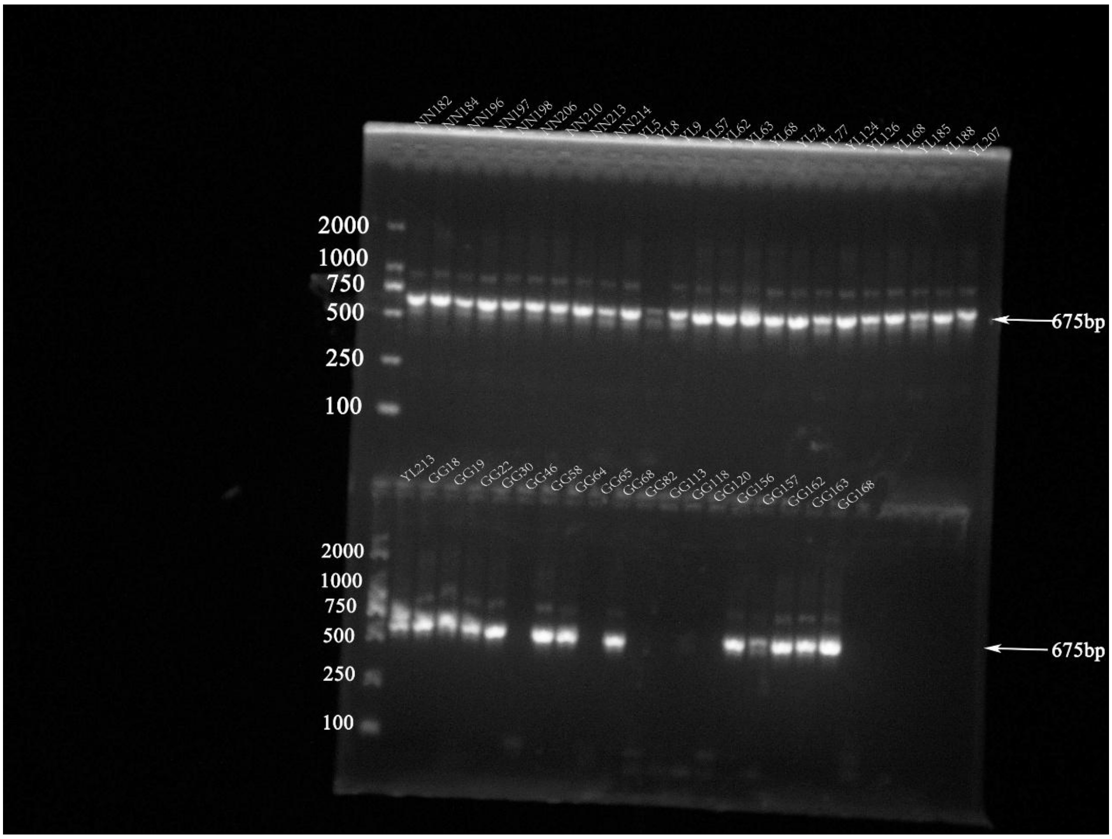
NN182
NN184
NN196
NN197
NN198
NN206
NN210
NN213
NN214
YL5
YL8
YL9
YL57
YL62
YL63
YL68
YL74
YL77
YL124
YL126
YL168
YL185
YL188
YL207
2000
1000
750
500
250
100
675bp
YL213
GG18
GG19
GG22
GG30
GG46
GG58
GG64
GG65
GG68
GG82
GG113
GG118
GG120
GG156
GG157
GG162
GG163
GG168
2000
1000
750
500
250
100
675bp

(c)MS3

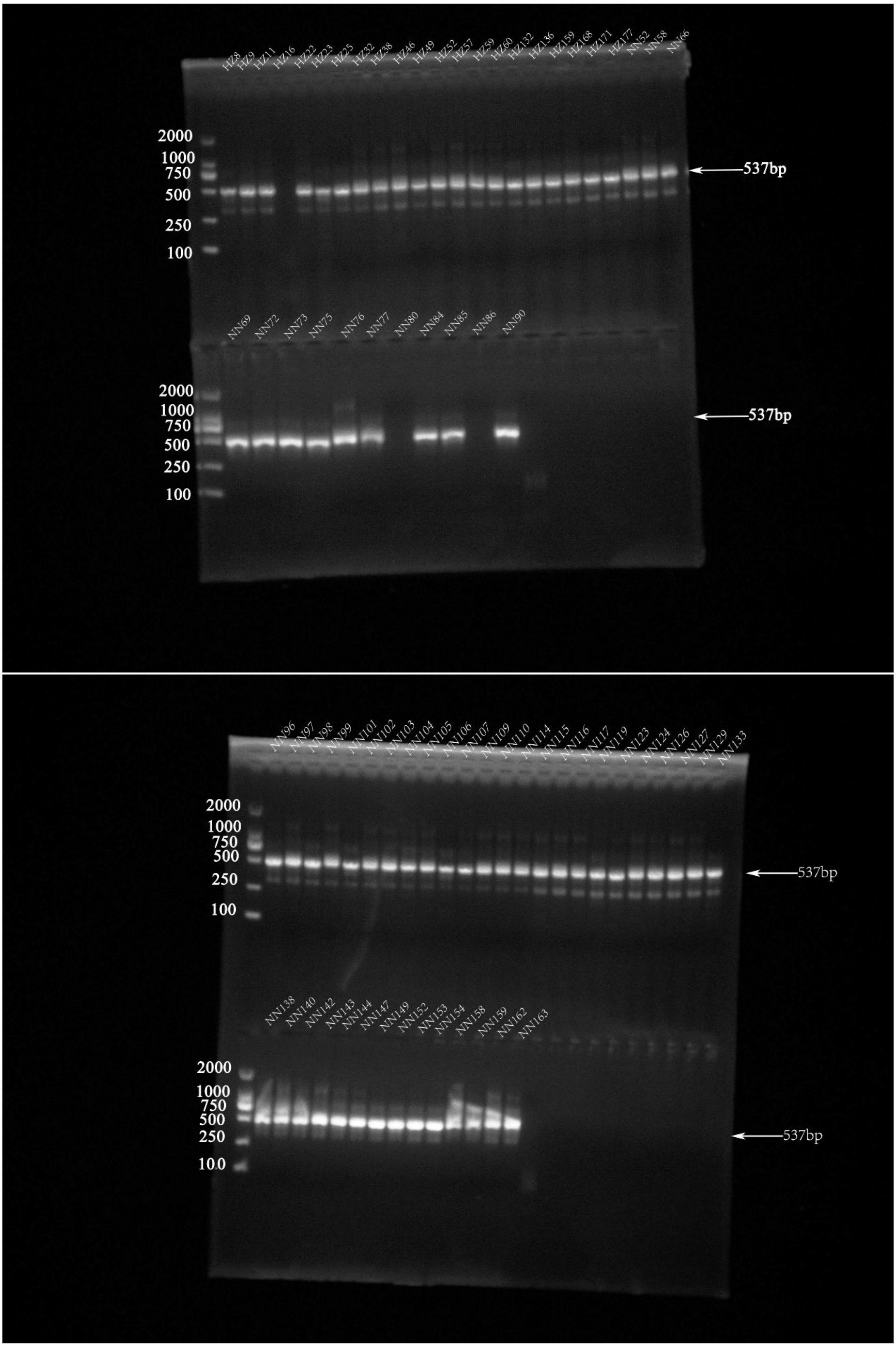
HZ8 HZ9 HZ11 HZ16 HZ22 HZ23 HZ25 HZ32 HZ38 HZ46 HZ49 HZ52 HZ57 HZ59 HZ60 HZ132 HZ136 HZ159 HZ168 HZ171 HZ177 NN52 NN58 NN66
2000
1000
750
500
250
100
537bp
NN69 NN72 NN73 NN75 NN76 NN77 NN80 NN84 NN85 NN86 NN90
2000
1000
750
500
250
100
537bp
NN96 NN97 NN98 NN99 NN101 NN102 NN103 NN104 NN105 NN106 NN107 NN109 NN110 NN114 NN115 NN116 NN117 NN119 NN123 NN124 NN126 NN127 NN129 NN133
2000
1000
750
500
250
100
537bp
NN138 NN140 NN142 NN143 NN144 NN147 NN149 NN152 NN153 NN154 NN158 NN159 NN162 NN163
2000
1000
750
500
250
10.0
537bp

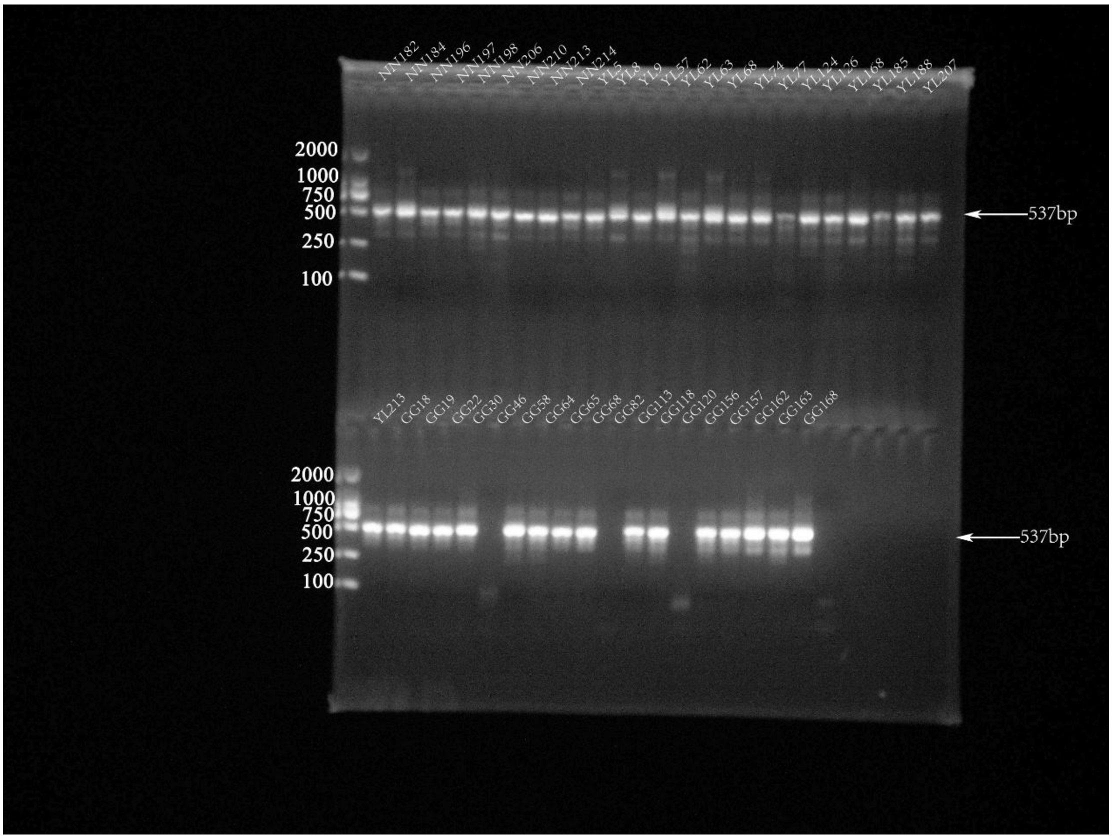
NN182
NN184
NN196
NN197
NN198
NN206
NN210
NN213
NN214
YL5
YL8
YL9
YL57
YL62
YL63
YL68
YL74
YL77
YL124
YL126
YL168
YL185
YL188
YL207
2000
1000
750
500
250
100
537bp
YL213
GG18
GG19
GG22
GG30
GG46
GG58
GG64
GG65
GG68
GG82
GG113
GG118
GG120
GG156
GG157
GG162
GG163
GG168
2000
1000
750
500
250
100
537bp

(d) MS4

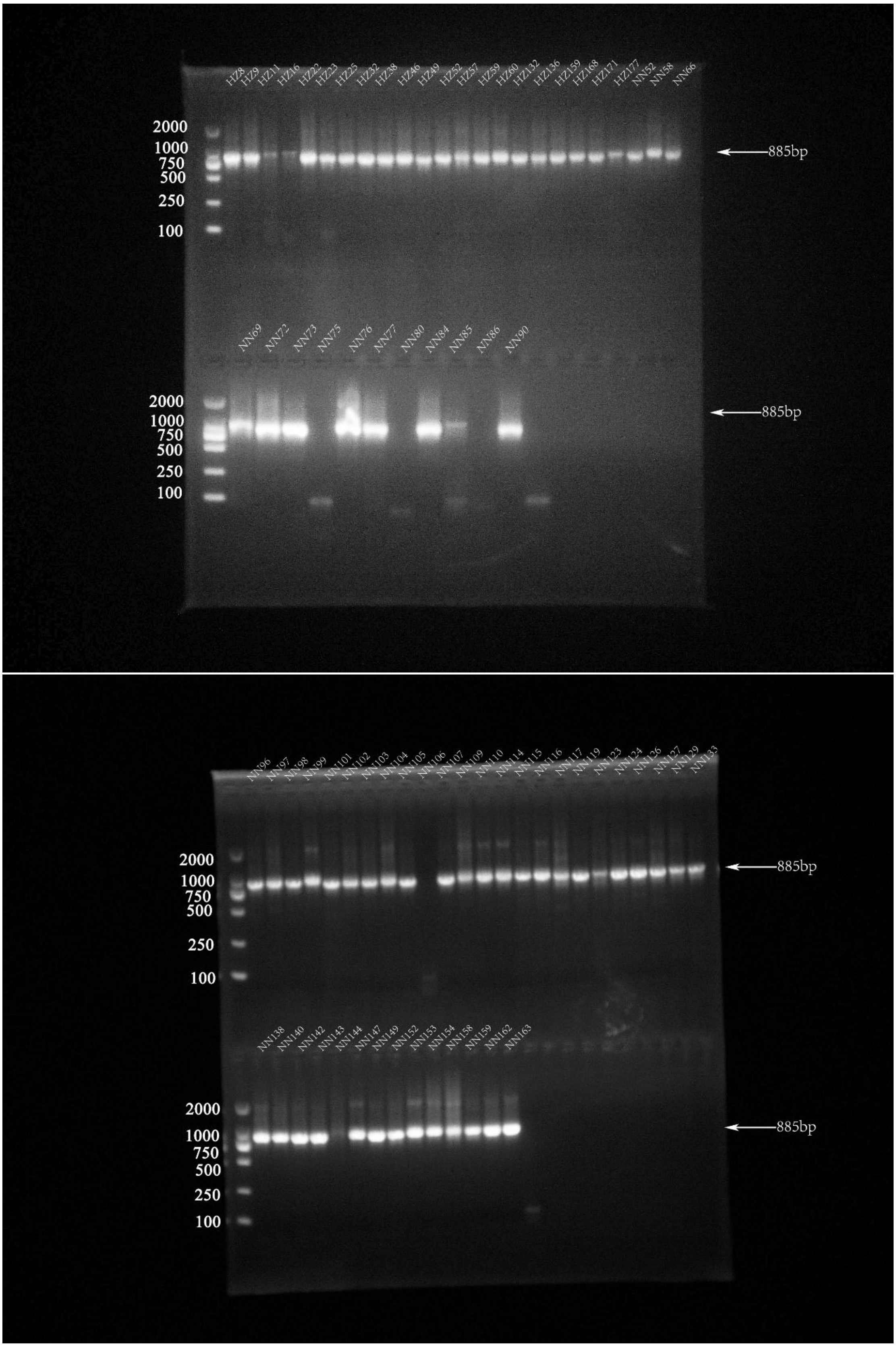

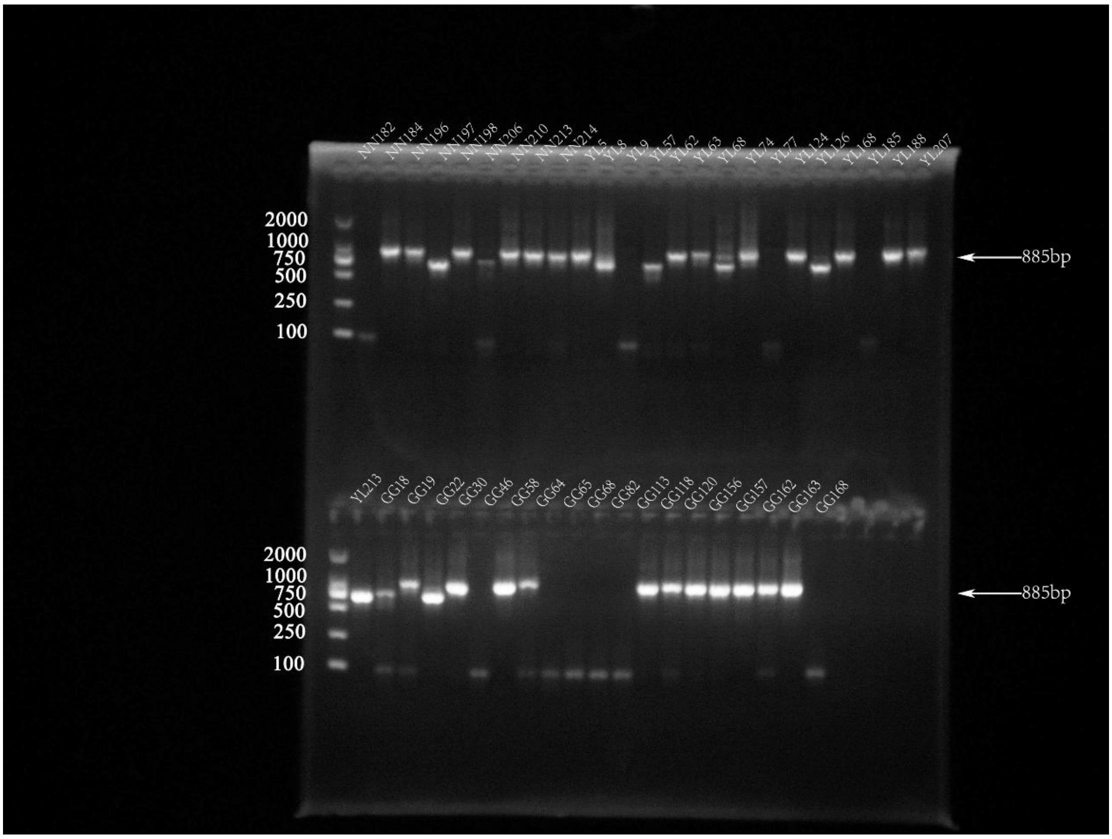
NN182
NN184
NN196
NN197
NN198
NN206
NN210
NN213
NN214
YL5
YL8
YL9
YL57
YL62
YL63
YL68
YL74
YL77
YL124
YL126
YL168
YL185
YL188
YL207
2000
1000
750
500
250
100
885bp
YL213
GG18
GG19
GG22
GG30
GG46
GG58
GG64
GG65
GG68
GG82
GG113
GG118
GG120
GG156
GG157
GG162
GG163
GG168
2000
1000
750
500
250
100
885bp

(e) MS7

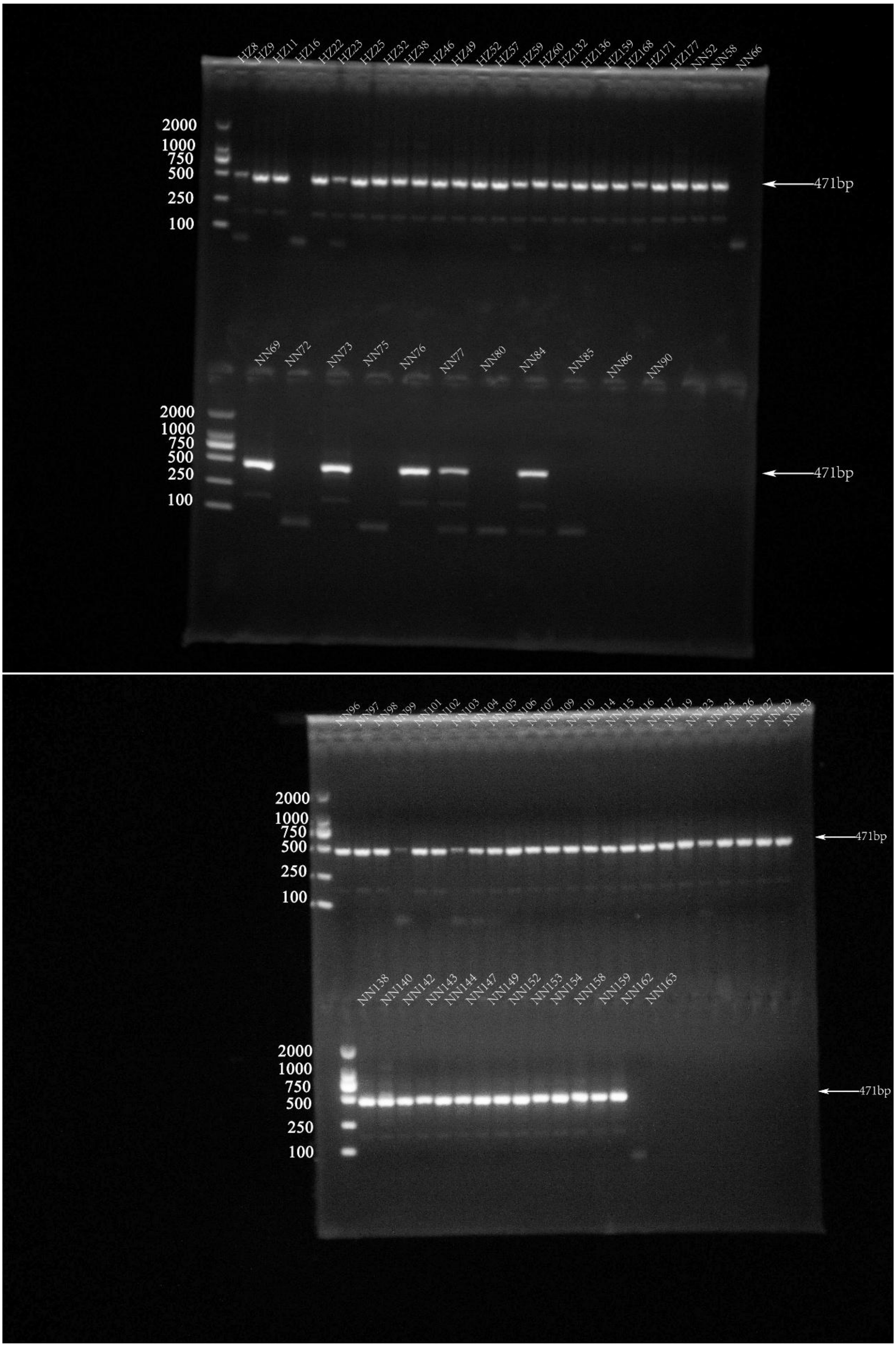
HZ8 HZ9 HZ11 HZ16 HZ22 HZ23 HZ25 HZ32 HZ38 HZ46 HZ49 HZ52 HZ57 HZ59 HZ60 HZ132 HZ136 HZ159 HZ168 HZ171 HZ177 NN52 NN58 NN66
2000
1000
750
500
250
100
471bp
NN69 NN72 NN73 NN75 NN76 NN77 NN80 NN84 NN85 NN86 NN90
2000
1000
750
500
250
100
471bp
2000
1000
750
500
250
100
471bp
NN138 NN140 NN142 NN143 NN144 NN147 NN149 NN152 NN153 NN154 NN158 NN159 NN162 NN163
2000
1000
750
500
250
100
471bp

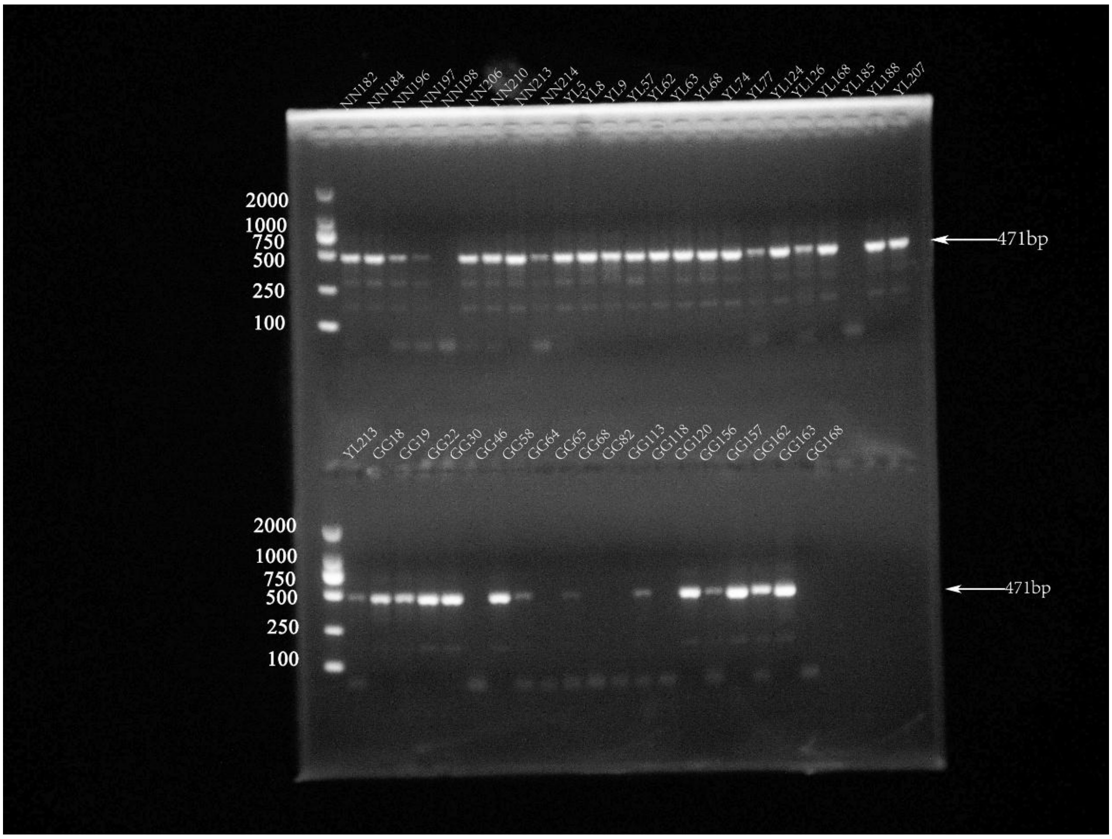
NN182
NN184
NN196
NN197
NN198
NN206
NN210
NN213
NN214
YL5
YL8
YL9
YL57
YL62
YL63
YL68
YL74
YL77
YL124
YL126
YL168
YL185
YL188
YL207
2000
1000
750
500
250
100
471bp
YL213
GG18
GG19
GG22
GG30
GG46
GG58
GG64
GG65
GG68
GG82
GG113
GG118
GG120
GG156
GG157
GG162
GG163
GG168
2000
1000
750
500
250
100
471bp
