# Supplementary material for: First molecular detection and multilocus genotyping of Enterocytozoon bieneusi from pigs in Guangxi Zhuang Autonomous region, Southern China
Source: BMC Vet Res. 2025 Jun 4;21:401. doi: 10.1186/s12917-025-04836-3 (PMC12135585; doi:10.1186/s12917-025-04836-3)
Supplement: Supplementary file 2 — Supplementary Material 2 [file 12917_2025_4836_MOESM2_ESM.docx]

**Table S1.** PCR primers (1A) and optimized reaction conditions (1B) for detection of *Enterocytozoon bieneusi*.

1A

| **Gene/Locus** | **Number** | **Sequence (5’-3’)** | **Ampliconsize (bp)** | **Reference** |
| --- | --- | --- | --- | --- |
| ITS | EBITS3-w-F | GGTCATAGAGTAGAGAGAGAGAG | ~435 | [1] |
|  | EBITS4-w-R | TTCGAGTTCTCTCGCGCGCTCTC |  |  |
|  | EBITS1-n-F | GCTCTGAATATCTATGGCTAG | 390 |  |
|  | EBITS2.4-n-R | ATCGCCGACGGATCCAAGTG |  |  |
| MS1 | MS1-w-F | CAAGTTGCAAGTTCAGTGTTTGAA | 843 | [2] |
|  | MS1-w-R | GATGAATATGCATCCATTGATGTT |  |  |
|  | MS1-n-F | TTGTAAATCGACCAAATGTGCTAT | 675 |  |
|  | MS1-n-R | GGACATAAACCACTAATTAATGTAAC |  |  |
| MS3 | MS3-w-F | GGACATAAACCACTAATTAATGTAAC | 702 |  |
|  | MS3-w-R | AAGTTAGGGCATTTAATAAAATTA |  |  |
|  | MS3-n-F | GTTCAAGTAATTGATACCAGTCT | 537 |  |
|  | MS3-n-R | CTCATTGAATCTAAATGTGTATAA |  |  |
| MS4 | MS4-w-F | CTCATTGAATCTAAATGTGTATAA | 1066 |  |
|  | MS4-w-R | GTTCATGGTTATTAATTCCAGAA |  |  |
|  | MS4-n-F | GTTCATGGTTATTAATTCCAGAA | 885 |  |
|  | MS4-n-R | GGACTTTAATAAGTTACCTATAGT |  |  |
| MS7 | MS7-w-F | GTTGATCGTCCAGATGGAATT | 684 |  |
|  | MS7-w-R | GACTATCAGTATTACTGATTATAT |  |  |
|  | MS7-n-F | CAATAGTAAAGGAAGATGGTCA | 471 |  |
|  | MS7-n-R | CGTCGCTTTGTTTCATAATCTT |  |  |

1B

| **Gene/Locus** |  | **Initial denaturation** | **Cycle** | **Denaturation** | **Annealed** | **Extension** | **Final extension** |
| --- | --- | --- | --- | --- | --- | --- | --- |
| ITS | ITS-w | 94°C/3 min | 35 | 94°C/40 s | 57°C/40 s | 72°C/60 s | 72°C/5 min |
|  | ITS-n | 95°C/3 min | 30 | 94°C/40 s | 55°C/40 s | 72°C/60 s | 72°C/5 min |
| MS1 | MS1-w | 94°C/5 min | 35 | 94°C/30 s | 58°C/30 s | 72°C/45 s | 72°C/5 min |
|  | MS1-n | 94°C/5 min | 35 | 94°C/30 s | 58°C/30 s | 72°C/40 s | 72°C/10 min |
| MS3 | MS3-w | 94°C/5 min | 35 | 94°C/30 s | 55°C/30 s | 72°C/45 s | 72°C/5 min |
|  | MS3-n | 94°C/5 min | 35 | 94°C/30 s | 55°C/30 s | 72°C/40 s | 72°C/10 min |
| MS4 | MS4-w | 94°C/5 min | 35 | 94°C/30 s | 55°C/30 s | 72°C/45 s | 72°C/5 min |
|  | MS4-n | 94°C/5 min | 35 | 94°C/30 s | 55°C/30 s | 72°C/40 s | 72°C/10 min |
| MS7 | MS7-w | 94°C/5 min | 35 | 94°C/30 s | 55°C/30 s | 72°C/45 s | 72°C/5 min |
|  | MS7-n | 94°C/5 min | 35 | 94°C/30 s | 55°C/30 s | 72°C/40 s | 72°C/10 min |

**References**

[1] Buckholt MA, Lee JH, Tzipori S. Prevalence of *Enterocytozoon bieneusi* in swine: an 18-month survey at a slaughterhouse in Massachusetts. Appl Environ Microbiol 2002;68(5):2595-2599.

[2] Feng Y, Li N, Dearen T, Lobo ML, Matos O, Cama V, Xiao L. Development of a multilocus sequence typing tool for high-resolution genotyping of *Enterocytozoon bieneusi*. Appl Environ Microbiol 2011;77(14):4822-4828.
