# Supplementary material for: First molecular detection and multilocus genotyping of Enterocytozoon bieneusi from pigs in Guangxi Zhuang Autonomous region, Southern China
Source: BMC Vet Res. 2025 Jun 4;21:401. doi: 10.1186/s12917-025-04836-3 (PMC12135585; doi:10.1186/s12917-025-04836-3)

Supplementary material 1.

PCR results of partial samples of ITS, MS1, MS3, MS4, and MS7 genes. M: Marker; P: Positive control; N: Negative control. (a) 1-7: Partial samples at ITS gene; (b) MS1 gene samples; (c) MS gene samples; (d) MS4 gene samples; (e) MS7 gene samples. sample information: Hezhou (HZ), Nanning (NN), Yulin (YL)， Guigang (GG).

(a) ITS

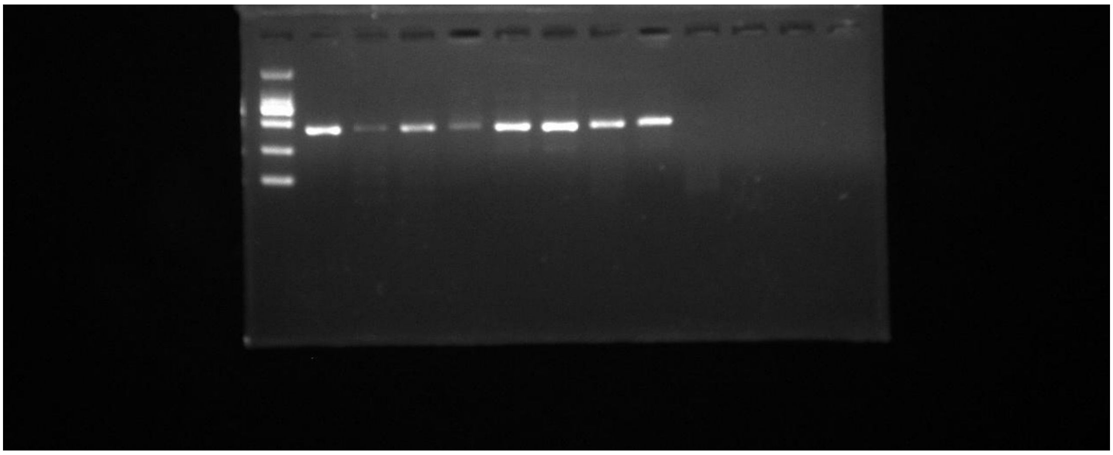

(b) MS1

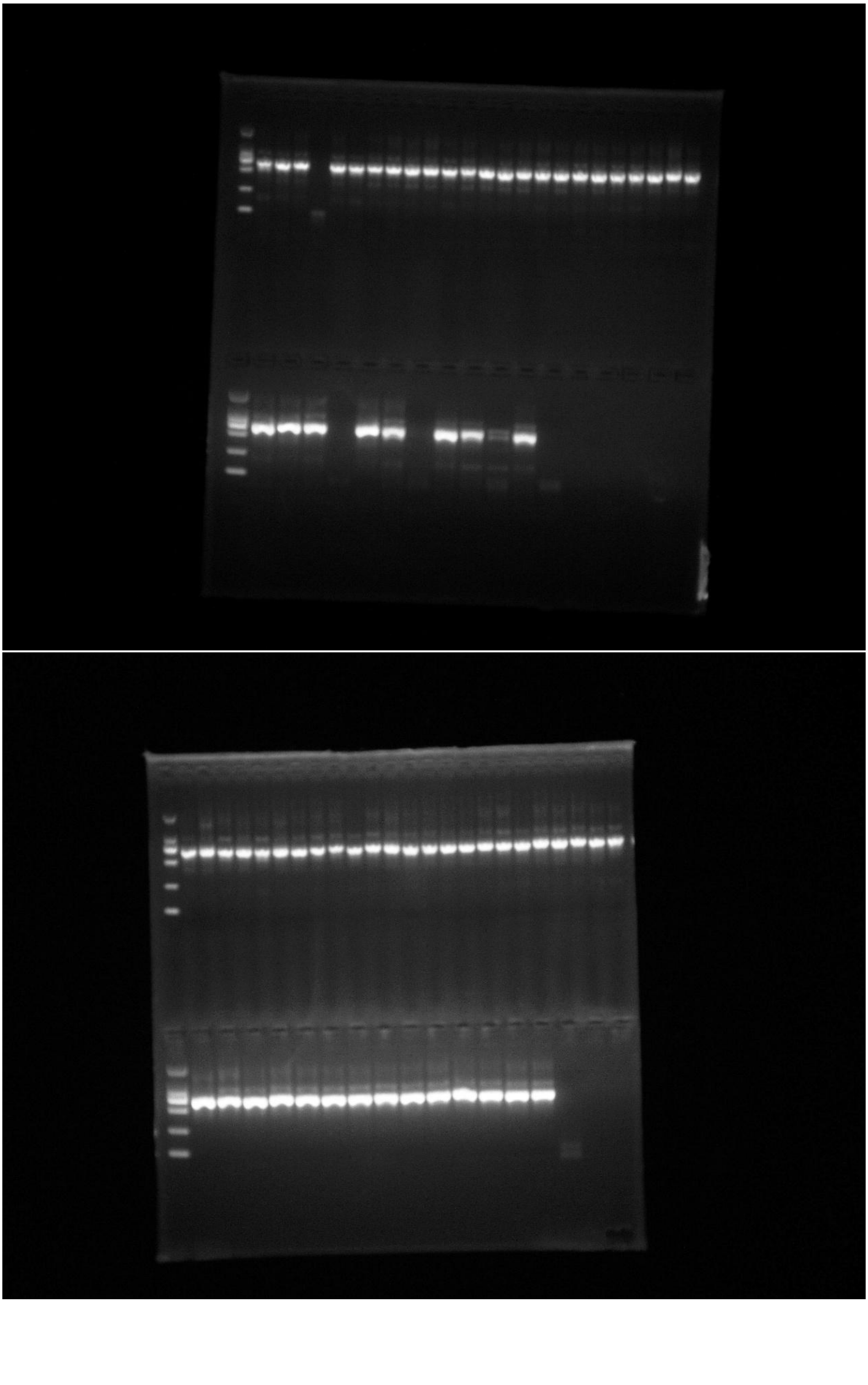

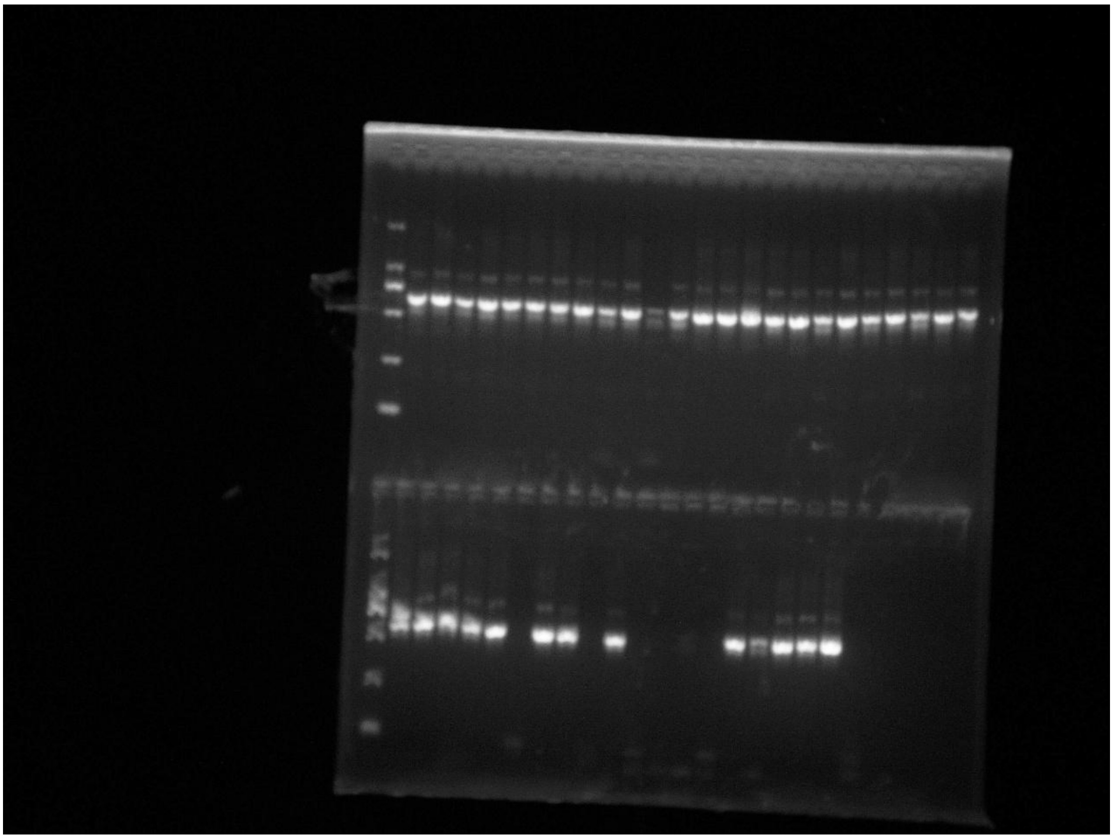

(c)MS3

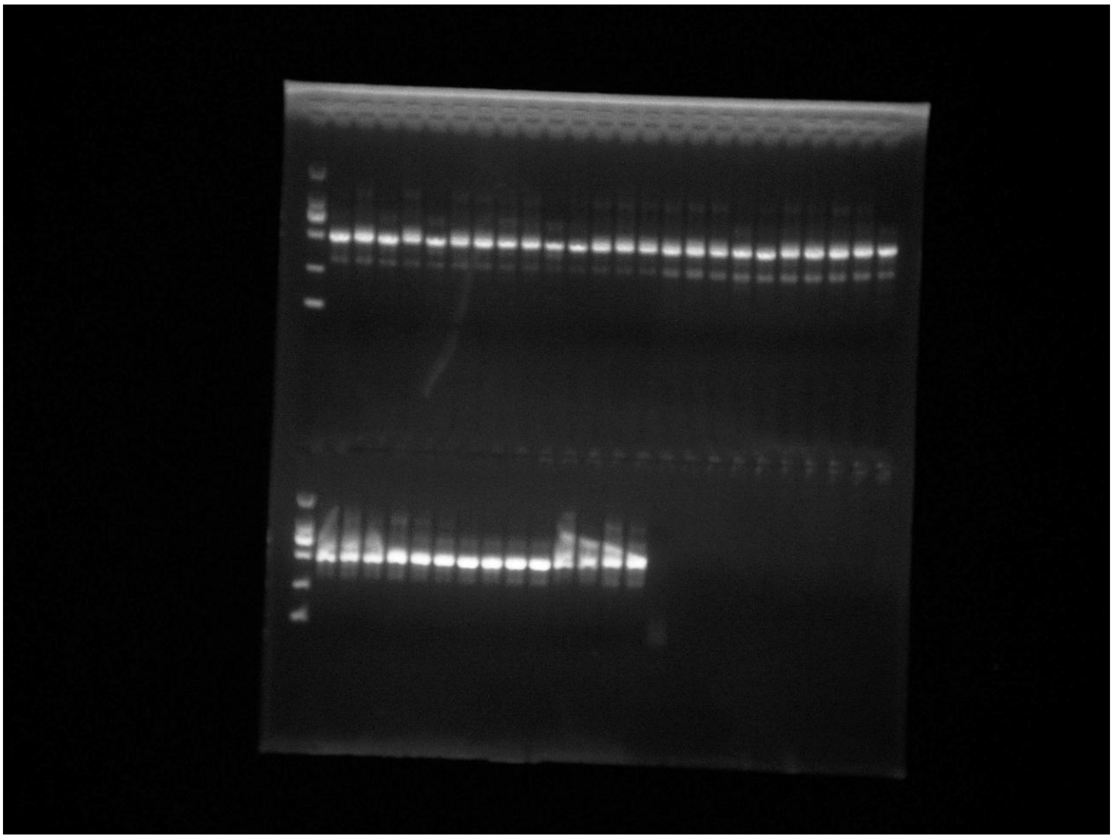

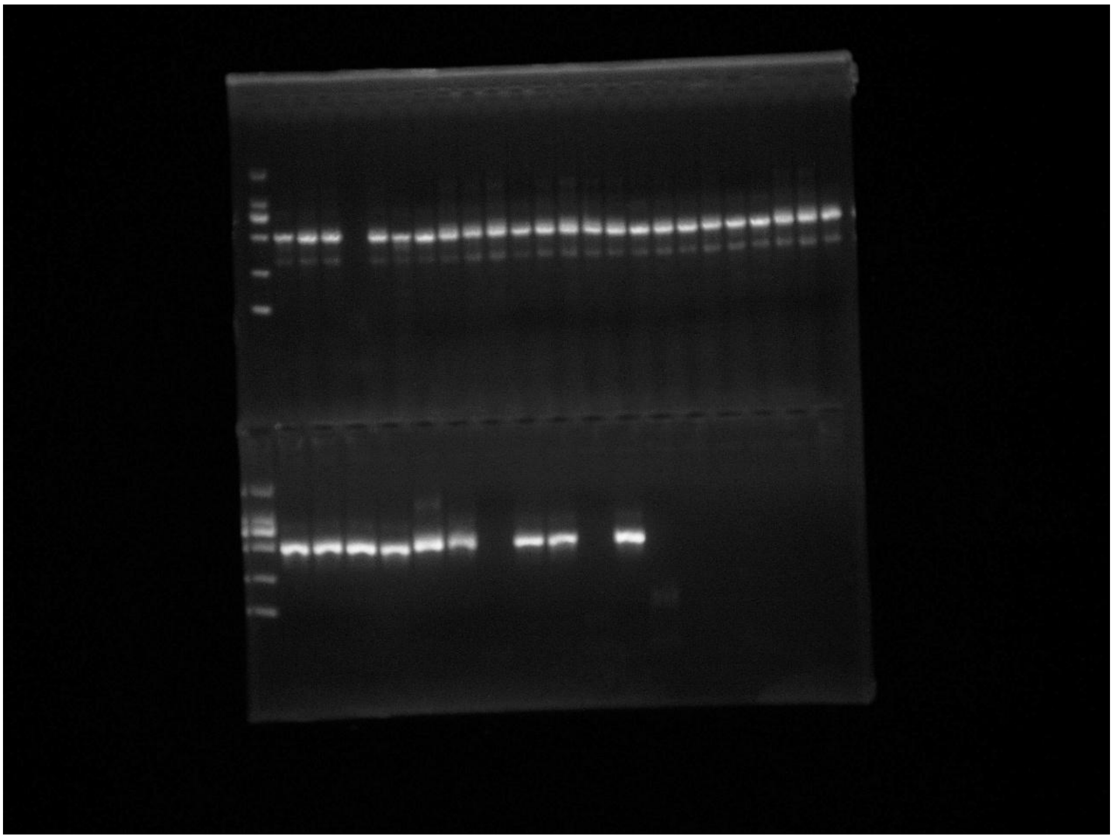

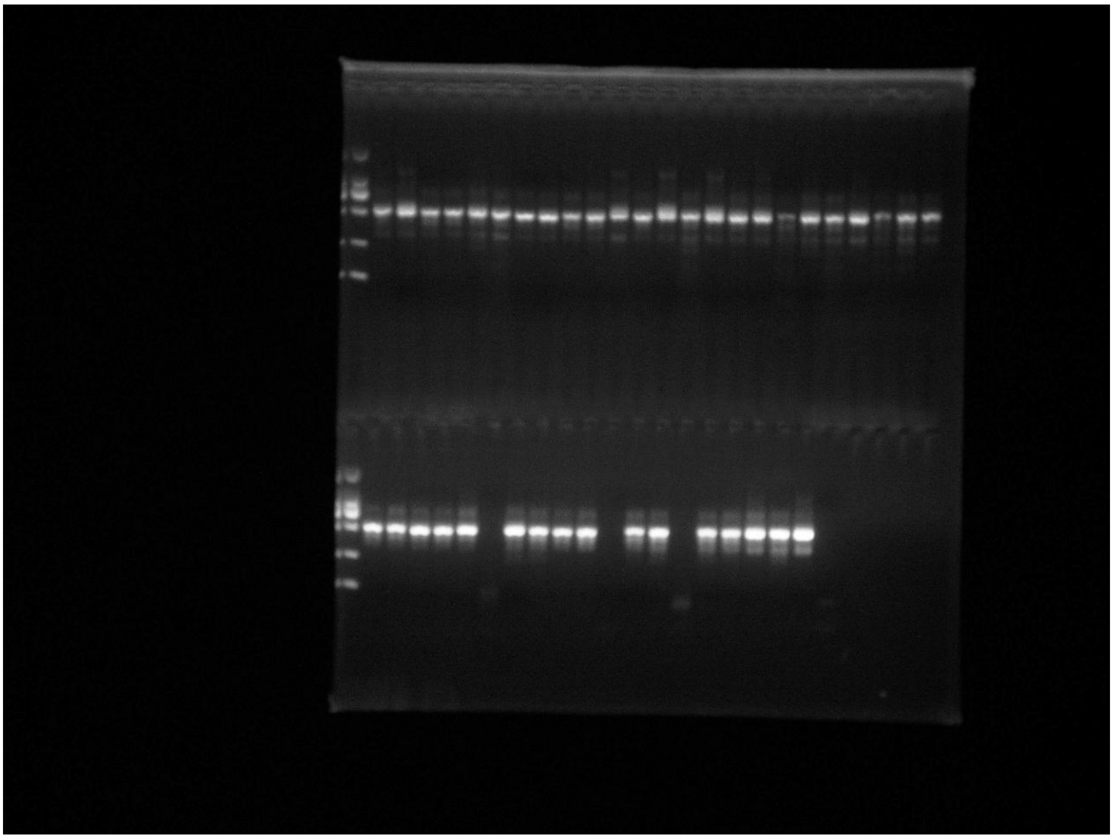

(d) MS4

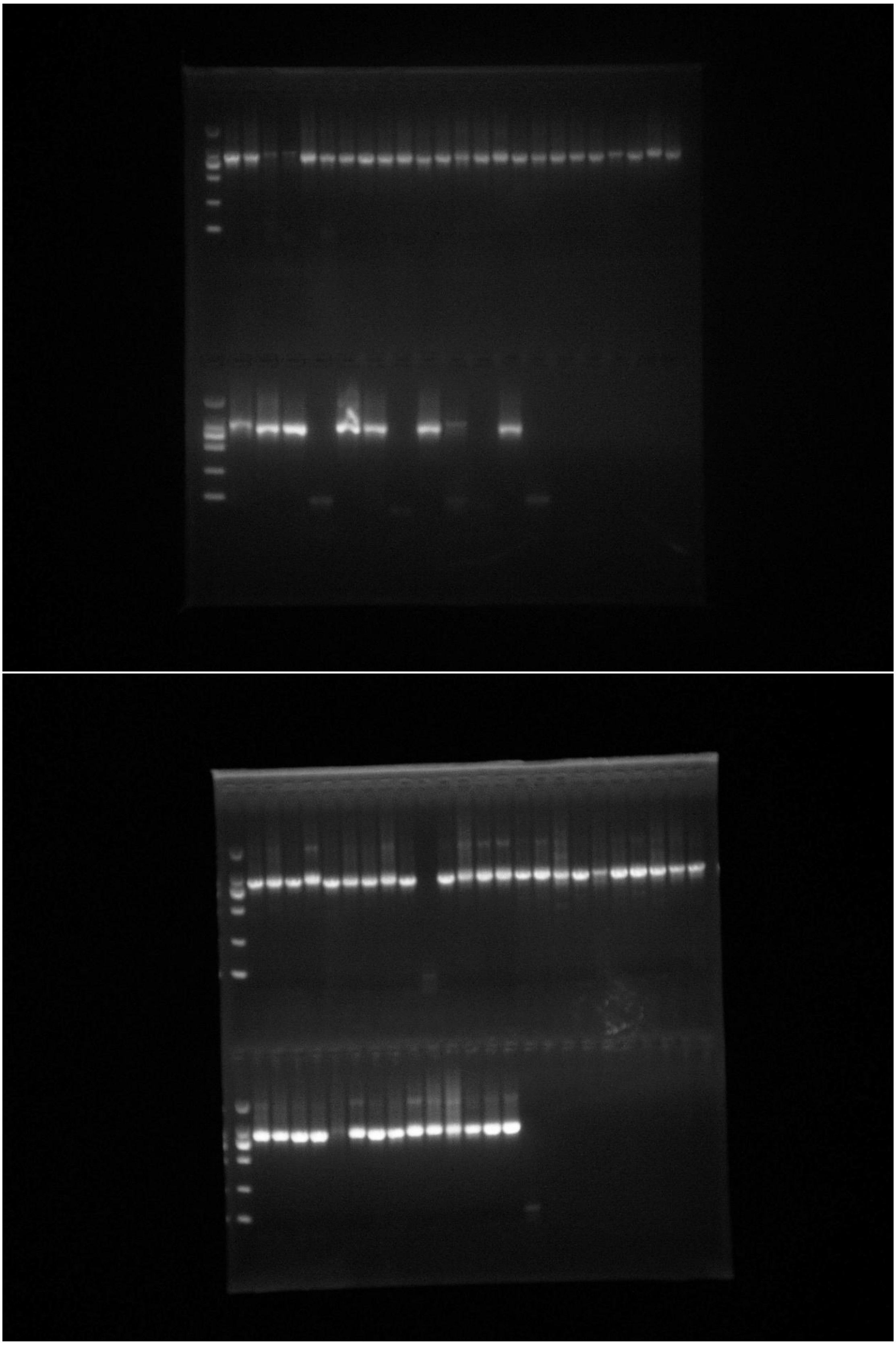

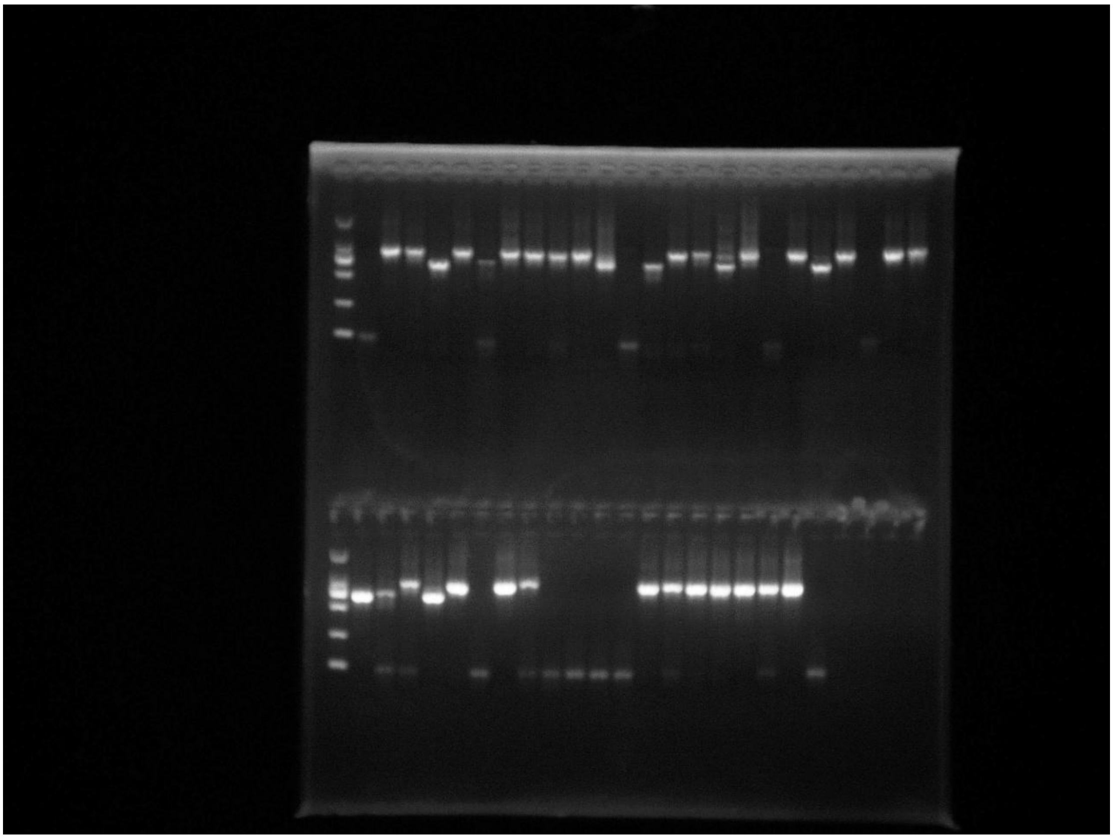

(e) MS7

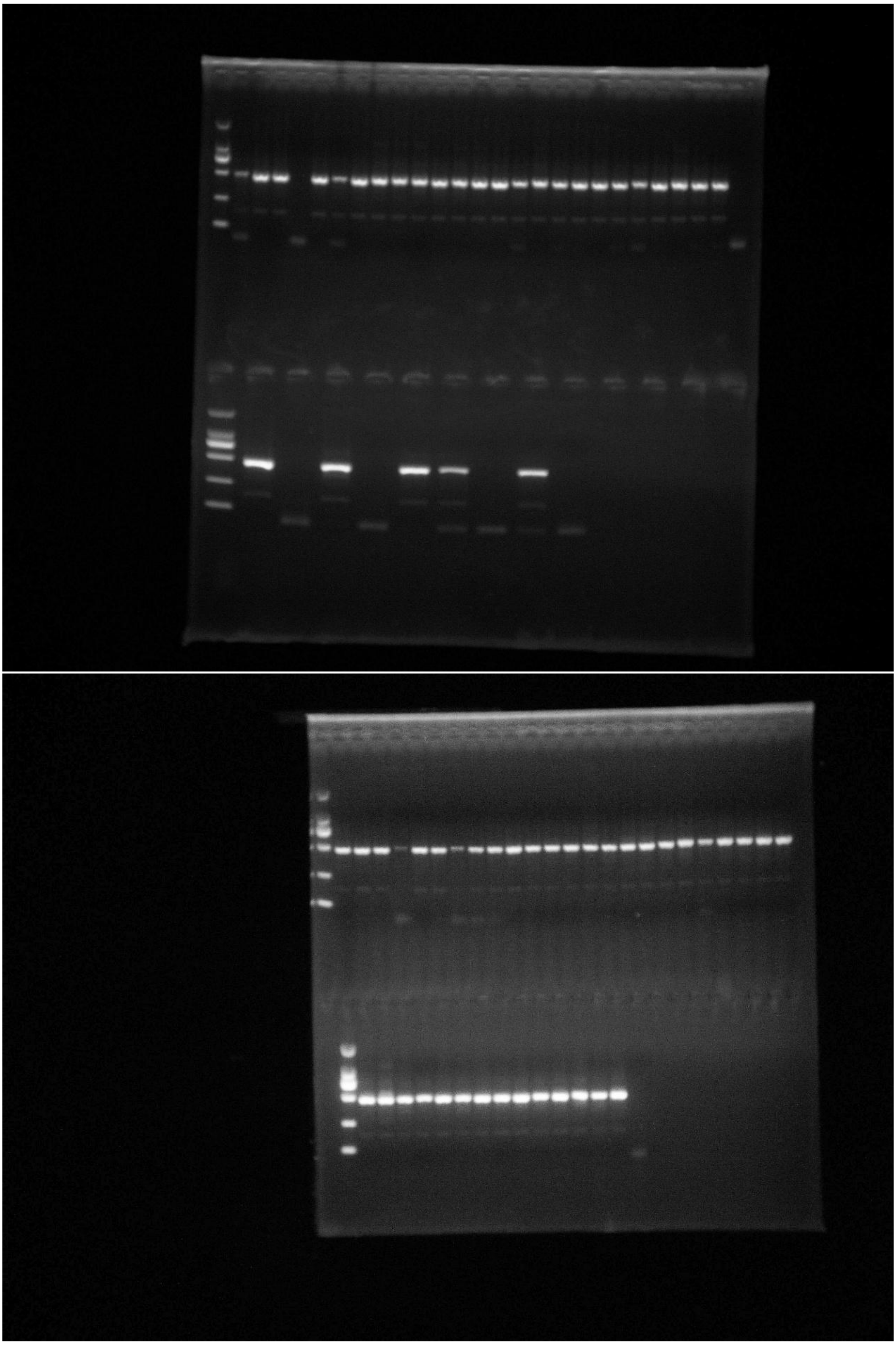

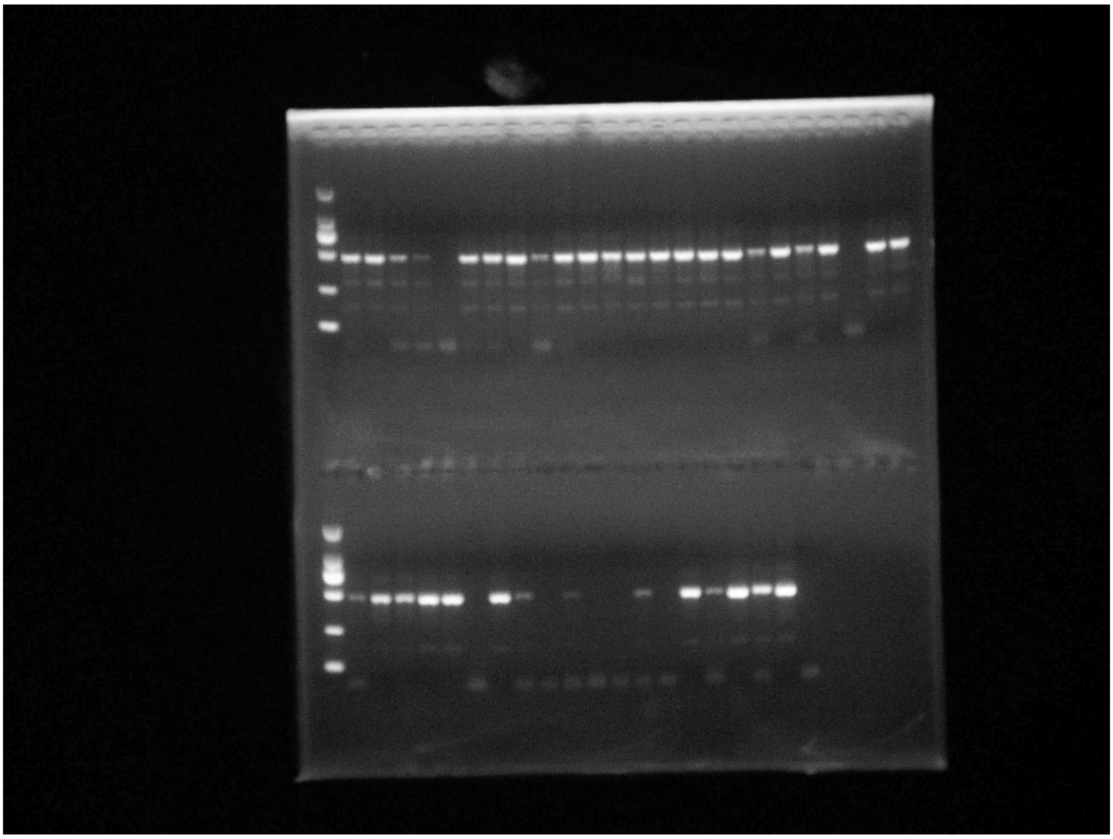

Supplement: Supplementary file 3 — Supplementary Material 3: File S1: Multilocus genotypes of E. bieneusi isolates from pigs [file 12917_2025_4836_MOESM3_ESM.pdf]
